# Thiol, His–His Motif, and the Battle over Cu(II) in the Relationship of CopM Metallophore and OprC Outer Membrane Protein

**DOI:** 10.1021/acs.inorgchem.4c05101

**Published:** 2025-02-06

**Authors:** Aleksandra Hecel, Arian Kola, Daniela Valensin, Danuta Witkowska

**Affiliations:** † Faculty of Chemistry, University of Wroclaw, 50-383 Wroclaw, Poland; ‡ Department of Biotechnology, Chemistry and Pharmacy, University of Siena, 53100 Siena, Italy; § Institute of Health Sciences, University of Opole, 45-060 Opole, Poland

## Abstract

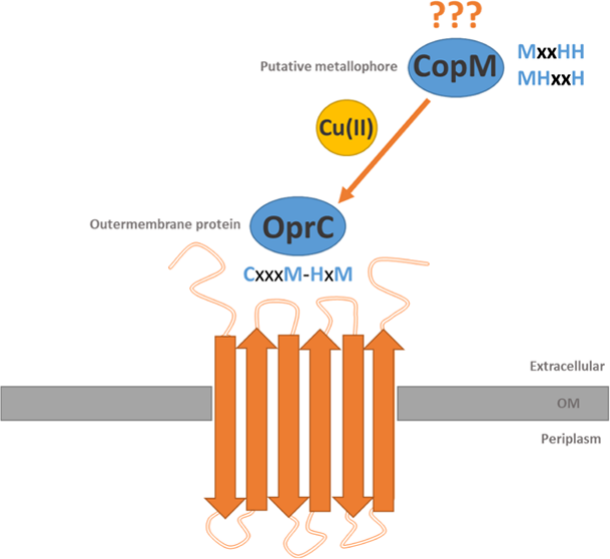

The mechanisms of Cu import across the bacterial outer membrane have been investigated only in a few cases. One such mechanism involves the outer membrane OprC transporter with a unique CxxxM-HxM metal-binding site, discovered recently. This newly identified site in OprC is located outside the cell and is, therefore, most likely to bind Cu(II) through this domain. Since OprC may interact with azurin to facilitate the removal of copper, our study investigated the potential role of CopM metallophore. We selected two putative metal-binding sites in CopM, characterized by MxxHH and MHxxH motifs, which can bind Cu(II) and may interact with the extracellular CxxxM-HxM motif of OprC. At pH 7, the MxxHH motif in CopM was the most effective ligand for Cu(II) ions compared to the MHxxH domain and the novel CxxxM-HxM site in OprC. Furthermore, the CxxxM-HxM site in OprC, where a cysteine residue also binds Cu(II) ions alongside histidine, does not effectively compete with the MxxHH metal-binding site in CopM. This comparison suggests that the CopM MxxHH domain binds Cu(II) ions very strongly and is unable to give them back to the OprC; therefore, it is perhaps transported together with copper ions through OprC into the bacterial cell.

## Abstract 2

The question is whether two metal-binding sites in CopM (MxxHH, MHxxH) may bind Cu(II) and interact with OprC’s CxxxM-HxM motif, aiding metal ion transport into bacterial cells

## Introduction

There is a significant gap in understanding copper import systems in prokaryotic organisms. Although the Ctr family transporters are quite well-described, the bacterial copper influx mechanism is very poorly understood, primarily because bacterial copper proteins tend to localize in the periplasmic and extracellular space.^1,2^ Furthermore, bacterial Cu transport systems are far more diverse than those in eukaryotes, and some copper uptake mechanisms are confined to one bacterial group or even one species; hence, understanding the transport mechanism is extremely complex. Studies on bacterial copper transport have mainly focused on copper export (in order to detoxify the microbial cell), while the mechanisms of copper import across the outer membrane of Gram-negative bacteria and the bacterial cytoplasmic membrane have been examined in only a few cases.^3,4^

Microorganisms are aware of the importance of the acquisition of substantial metal trace elements. Transition metals are significant for the survival of all organisms, being crucial for metalloproteins, such as metalloenzymes, storage proteins, and transcription factors. Pathogens have developed highly efficient transport systems, which rely on (i) chaperones, metal-chelating molecules present in microorganisms in order to efficiently bind metal ions and (ii) transmembrane transporters (importer and exporter) that interact with specific chaperones and are capable of transporting metal ions into the periplasm and cytoplasm. The effective acquisition of metal ions is often considered as a virulence factor and is thus a potential target for novel antimicrobial therapies.^5^ In general, the copper transport system in Gram-negative bacteria is more complex than that in Gram-positive ones due to the presence of the periplasmic space. It includes (i) transmembrane proteins, which are responsible for capturing and transporting copper ions to or from the periplasm and cytosol (e.g., CcoA, OprC, CuiT, CopA, CusABC) and (ii) peptide chaperones in the periplasmic and cytoplasmic space (e.g., PcoC, CopZ, CusF), whose role is to deliver copper to specific importer/exporter anchored in the membrane and/or deliver copper ions to other proteins (e.g., enzymes).^6,7^ This very complex system is also completed by sensors regulating the bioavailability of Cu(I) in the cell compartment and storage copper proteins. Depending on whether a given transporter is localized outside or inside the cell, it can bind copper ions in different oxidation states.

Only few copper influx proteins have been identified in bacteria so far—the outer membrane, OprC, and the inner-membrane transporters, CcoA^8^ and CuiT.^9^ OprC (*P00282, UniProt*) is an outer membrane, TonB-dependent transporter that is conserved in many *Proteobacteria* and which mediates acquisition of both reduced and oxidized ionic copper.^10−13^ It binds Cu(II)/Cu(I) via an unprecedented CxxxM-HxM metal-binding site, discovered recently.^12^ Furthermore, the blue copper protein azurin was reported to be secreted by *Pseudomonas aeruginosa* and to interact with OprC, suggesting a role of the latter in Cu(II) uptake. There is not much information in the literature about copper-binding peptide metallophores identified in bacteria. The copper resistance system in the cyanobacterium *Synechocystis* comprises two operons, previously described copBAC, which are expressed in response to copper in the media, and copMRS, which codes for a protein of unknown function. One of them is CopM (*A0A0F6QDN6, UniProt)* protein, which is able to bind both Cu(I) and Cu(II).^14,15^ It is worth noting that CopM was localized not only in the periplasm but also in the extracellular space. It suggests that CopM can act not only as chaperone but also as a metallophore, excreting outside the pathogen in order to efficiently bind copper ions. This can serve either as metal acquisition or as a way of protecting the atoms against the toxic excess of metal ions. CopM involves a DUF305 domain of unknown function with a characteristic _53_MxxHH_57_ motif, located in the first α-helical domain.^16^ The Cu(II)–CopM structure from crystals soaked with Cu(II) was determined, and residues 114–125 of the linker region can be traced in the electron-density map. In this region, two histidine residues may constitute the copper-binding site, with the _117_MHxxH_121_ motif.^15^

In this study, we focus on Cu(II) complexes involving a novel CxxxM-HxM metal-binding site from an OprC transmembrane protein. This newly identified OprC site is localized outside the cell, and therefore, it is most likely to bind Cu(II) through this domain. The Ac-_141_GA**C**PNR**M**DA_149_AAAAAA_321_AD**H**I**M**D_326_-NH_2_ sequence was synthesized with two binding domains connected with a 6Ala linker. Due to the fact that OprC may interact with azurin to effectively withdraw copper ions, our study also focused on the potential CopM metallophore. We have chosen two putative Cu(II) binding sites from the CopM amino acid sequence (Ac-_51_EM**M**TP**HH**QDAIDMAEMALQKAEHPE_75_-NH_2_ and Ac-_113_GMMG**MH**QG**H**GMMAMD_127_-NH_2_), which contain MxxHH and MHxxM motifs, respectively, capable of binding Cu(II) and can possibly interact with the extracellular CxxxM-HxM OprC domain to convey a metal ion into the bacterial cell. Potentiometric titrations were used to calculate the thermodynamic parameters of the investigated systems. We determined the partial and overall stability constants for all formed Cu(II) complexes. In order to precisely establish metal-binding sites, donor atoms involved in the coordination sphere, and the coordination geometry of the formed complexes, several spectroscopic techniques—UV–vis, circular dichroism (CD), NMR and electron paramagnetic resonance (EPR) measurements—were registered at different pH values.

## Experimental Section

### Materials

Three peptides, Ac-GA**C**PNR**M**DAAAAAAAAD**H**I**M**D-NH_2_, Ac-EM**M**TP**HH**QDAIDMAEMALQKAEHPE-NH_2_, and Ac-GMMG**MH**QG**H**GMMAMD-NH_2_, were purchased from Karebay Biochem, with a certified purity of 98%, and used as received. Cu(II) perchlorate, obtained from Sigma-Aldrich, was of extra pure grade. A carbonate-free stock solution of 0.1 M KOH was also purchased from Sigma-Aldrich and subsequently standardized potentiometrically using potassium hydrogen phthalate.

### Potentiometric Measurements

The stability constants for proton and Cu(II) complexes with three ligands were determined from titration curves obtained over a pH range of 2–11, at 298 K and at an ionic strength of 0.1 M NaClO_4_. Each titration used a total solution volume of 3.0 cm^3^. The potentiometric titrations were conducted using a Dosimat 800 Metrohm Titrator paired with a Metrohm 905 pH meter and a Mettler Toledo pH InLab Science electrode. The titration setup included a thermostabilized glass cell with magnetic stirring, a microburet delivery tube, and an argon inlet–outlet tube. Titrations were performed with 0.1 M carbonate-free NaOH. Daily calibration of the electrodes for hydrogen ion concentration was achieved by titrating HClO_4_ with NaOH in a 3.0 cm^3^ solution. Ligand concentrations were maintained at 0.4 and 0.5 mM, with metal-to-ligand ratios at 1:1. The concentrations and purities of the ligand solutions were verified by using the Gran method. The standard potential and electrode slope were calculated using the GLEE program.^17^ Stability constants were calculated with the HYPERQUAD 2008 software^18^ and speciation diagrams were generated using the HYSS program.^19^

### Spectroscopic Studies

Absorption spectra were recorded using a Jasco-730 spectrophotometer over the range 200–800 nm, with a 1 cm optical path length quartz cuvette. Circular dichroism (CD) spectra were measured on a Jasco J-1500 CD spectrometer within the 200–800 nm range, utilizing a quartz cuvette with a 1 cm and 0.01 mm optical path for the visible and near-UV range. The sample solution concentrations used in the spectroscopic studies were consistent with those used in the potentiometric experiments, maintaining a metal-to-ligand ratio of 1:1. All spectroscopic measurements were recorded in the pH range 3–11. The pH of the samples was adjusted with the appropriate amounts of HClO_4_ and NaOH solutions. The UV–vis and CD spectroscopy parameters were calculated from the spectra obtained at pH values corresponding to the maximum concentration of each particular species based on distribution diagrams. The spectra were processed and visualized using OriginPro 2016. Electron paramagnetic resonance (EPR) spectra were recorded in liquid nitrogen on a Bruker ELEXSYS E500 CW-EPR spectrometer at X-band frequency (frequency = 9.5 GHz, modulation amplitude = 10.00 G) and equipped with an ER 036TM NMR teslameter and an E41 FC frequency counter. Ethylene glycol (25%) was used as a cryoprotectant. Ligand concentration was 1 mM and Cu(II)-to-ligand ratio = 1:1. The EPR parameters were analyzed by simulating the experimental spectra using WIN-EPR SIMFONIA software, version 1.2 (Bruker).

### Mass Spectrometry

High-resolution mass spectra were acquired by using a Bruker compact QTOF mass spectrometer (Bruker Daltonik, Bremen, Germany) with an electrospray ionization source featuring an ion funnel. The instrument operated in positive ion mode with the following settings: scan range of *m*/*z* 100–2000, nitrogen as the dry gas, a temperature of 453 K, and ion energy of 5 eV. The capillary voltage, optimized for the highest signal-to-noise ratio, was set at 4800 V. Samples were prepared in a 1:1 methanol-to-water mixture (MeOH) at pH 6–7, with a metal-to-ligand molar ratio of 1:1 and a total ligand concentration of 0.1 mM. Infusion was carried out at a flow rate of 3 μL/min. External calibration of the instrument was performed using a Tunemix mixture (Bruker Daltonik, Germany) with quadratic regression. Data analysis was conducted with Compass DataAnalysis 4.2 software (Bruker Daltonik, Germany). Calibration accuracy was better than 5 ppm, allowing for unambiguous confirmation of the elemental composition of the complex using the true isotopic pattern and SigmaFit.

### NMR

All NMR experiments were conducted at a magnetic field strength of 14.1 T using a Bruker Avance III 600 MHz spectrometer, with the temperature maintained at 288 K. A 5 mm broadband inverse (BBI) probe was employed for all measurements. The residual water signal was suppressed through excitation sculpting, utilizing a selective square pulse of 2 ms duration targeting water.^20^ A typical NMR spectrum was recorded with 16 transients, a spectral width of 7200 Hz, and a recycle delay of 2.0 s. Proton resonance assignments for all of the peptide sequences were made using two-dimensional (2D) ^1^H–^1^H TOCSY and NOESY experiments. The TOCSY experiment was performed with a 60 ms spin-locking period by using the MLEV-17 mixing sequence. NOESY spectra were acquired at various mixing times to optimize the conditions. Data processing was completed using TopSpin v 3.6 software.

### Isothermal Titration Calorimetry (ITC)

Isothermal titration calorimetry (ITC) experiments were conducted at 25 °C using a MicroCal PEAQ titration calorimeter from Malvern, U.K. All reagents were of high purity (>99%) and obtained from Sigma-Aldrich. The peptides were dissolved directly in sodium cacodylate (Caco) buffer solution, pH 7.0. A metal stock solution of copper(II) ions was prepared at a concentration of 100 mM in deionized water (18 MΩ) at a low pH (∼2). Working solutions of copper(II) ions (1–2 mM) in Caco buffer were then prepared by dilution, with the pH adjusted to 7.0 using either NaOH or HCl.

Prior to titration, the instrument was stabilized at 25 °C. A total of 40 μL of the metal-buffer solution (1–2 mM) was used to titrate 200 μL of the peptide buffer solution, which initially had a concentration 10 times lower than that of the metal ion. Each assay was repeated at least three times. Each titration comprised 19 successive injections, with intervals of 180–240 s between each aliquot (depending on the time required for complete equilibration) and a stirring speed of 750 rpm.^21^ Additionally, a competitive reaction was conducted in which the Ac-EM**M**TP**HH**QDAIDMAEMALQKAEHPE-NH_2_ peptide (at a concentration of 1 mM) was titrated into the Ac-GA**C**PNR**M**DAAAAAAAAD**H**I**M**D-NH_2_ peptide (0.1 mM) that was already bound to copper ions, maintaining a 1:1 ratio.

The heat of dilution from an analogous control titration was subtracted prior to data fitting, and an initial injection of 0.4 μL was discarded from each data set to negate the effect of titrant diffusion across the syringe tip during the equilibration phase. Periodic titrations with CaCl_2_–EDTA were performed for comparison with the results obtained during the initial calibration of the instrument. Data analysis was performed using MicroCal PEAQ-ITC Analysis Software. The one-site binding model yielded the best-fit values for stoichiometry (*N*), enthalpy change (Δ*H*), and equilibrium constant (*K*_d_).

## Results and Discussion

### Ligand Protonation Constants of OprC and CopM

All peptides are protected in the N-terminus through acetylation and in the C-terminus through amidation.

The OprC fragment Ac-GA**C**PNR**M**DAAAAAAAAD**H**I**M**D-NH_2_ contains three Asp residues, which can release a proton from their carboxylic side chain. In addition to these acidic residues, the peptide has one histidine and one cysteine residue, which participate in acid–base equilibria through the imidazole ring and thiol group of their side chains, respectively (Table 1A). The release of acidic protons from Asp residues occurs with a dissociation constant of 2.53–4.46. The p*K*_a_ values of the side imidazole group of histidine (6.92) and thiol group of cysteine (9.56) are in agreement with literature values. For the metal-binding domain of CopM with the _53_MxxHH_57_ motif, Ac-EM**M**TP**HH**QDAIDMAEMALQKAEHPE-NH_2_, 11 protonation constants were detected (Table 1B). The six lowest constants (p*K*_a_ = 3.11–5.14) correspond to deprotonation of the carboxylic group of aspartic and glutamic acid residues. According to the literature,^22^ the two lower values were assigned to aspartic acid, which is generally more acidic than glutamic acid. The next three deprotonation constants (p*K*_a_ = 6.18, 6.86, and 7.43) can be assigned to the three imidazole groups from side chains of histidine residues. The highest p*K*_a_ = 9.94 belongs to the ε amine group of side chains of the lysine residue.

**Table 1:** Protonation Constants for (A) Ac-GACPNRMDAAAAAAAADHIMD-NH_2_, (B) Ac-EMMTPHHQDAIDMAEMALQKAEHPE-NH_2_, and (C) Ac-GMMGMHQGHGMMAMD-NH_2_, *T* = 298 K, *I* = 0.1 M (NaClO_4_)^a^

	log β*_jk_*^b^	p*K*_a_^c^	aa
(A) Ac-GACPNRMDAAAAAAAADHIMD-NH_2_			
[H_5_L]^+^	27.02(3)	2.53	D
[H_4_L]	24.49(3)	3.55	D
[H_3_L]^−^	20.94(3)	4.46	D
[H_2_L]^2–^	16.48(3)	6.92	H
[HL]^3–^	9.56(3)	9.56	C
(B) Ac-EMMTPHHQDAIDMAEMALQKAEHPE-NH_2_			
[H_10_L]^4+^	55.35(6)	3.11	D
[H_9_L]^3+^	52.24(4)	3.58	D
[H_8_L]^2+^	48.66(3)	4.04	E
[H_7_L]^+^	44.62(3)	4.34	E
[H_6_L]	40.28(3)	4.73	E
[H_5_L]^−^	35.55(2)	5.14	E
[H_4_L]^2–^	30.41(2)	6.18	H
[H_3_L]^3–^	24.23(2)	6.86	H
[H_2_L]^4–^	17.37(2)	7.43	H
[HL]^5–^	9.94(1)	9.94	K
(C) Ac-GMMGMHQGHGMMAMD-NH_2_			
[H_3_L]^2+^	16.76(2)	3.80	D
[H_2_L]^+^	12.96(2)	5.95	H
[HL]	7.01(2)	7.01	H

a Values in parentheses are standard deviations of the last significant figure.

b Constants are presented as cumulative log β*_jk_* values. β(*H_j_L_k_*) = [*H_j_L_k_*]/([*H*]*_j_*[*L*]*_k_*), in which [*L*] is the concentration of the fully deprotonated peptide.

c p*K*_a_ values of the peptides were derived from cumulative constants: p*K*_a_ = log β(*H_j_L_k_*) – log β(*H_j_* – 1*L_k_*).

For the CopM fragment, which contains the _117_MHxxH_121_ motif, Ac-GMMG**MH**QG**H**GMMAMD-NH_2_, 3 groups are involved in acid–base reactions: the aspartic acid side chain carboxylate and two histidine imidazoles with p*K*_a_ values 3.80, 5.95, and 7.01, respectively (Table 1C). All of the obtained protonation constants log β and calculated p*K*_a_ values for three ligands are in good agreement with the literature data for similar peptide systems.^23−26^

### OprC and CopM Copper(II) Complexes

The structural and thermodynamic properties of OprC and CopM-fragment copper complexes (the M/L stoichiometric ratio of 1:1) were studied using mass spectrometry, potentiometry, UV–vis, CD, NMR spectroscopy, and ITC.

Mass spectrometry revealed the stoichiometry of the copper complexes, showing that under the discussed conditions, only monometallic 1:1 Cu/L species exist in the solution for three systems. The MS spectra (Figure S1) and table (Table S1) with *m*/*z* values for three peptides and their copper complexes are presented in the Supporting Information. The *m*/*z* signals of free ligands and their Cu(II) complexes were confirmed by the analysis of the isotopic distribution of their signals and by the comparison of the experimental signals with those of their simulations.

**Figure 1:**
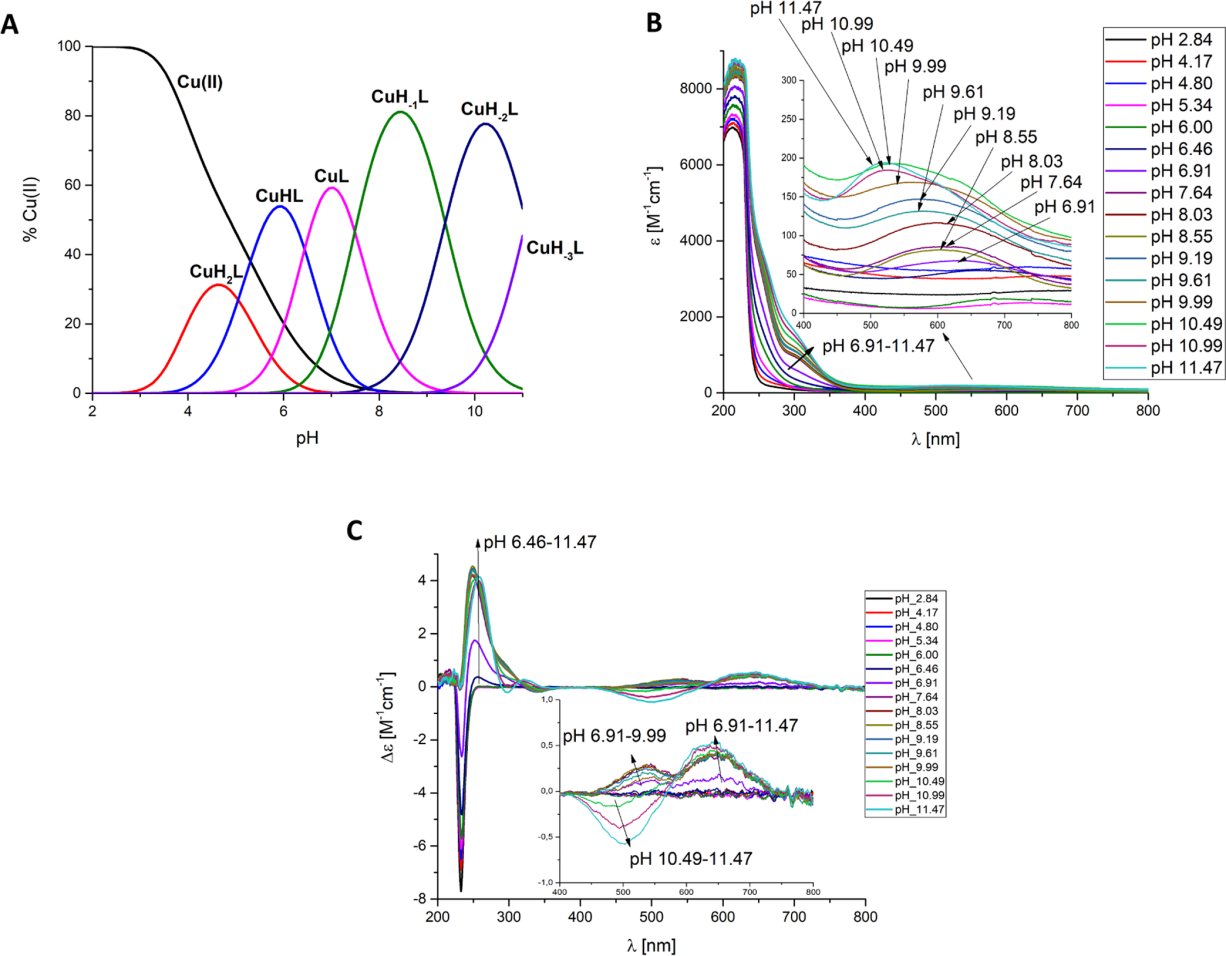
(A) Species distribution diagram, (B) UV–vis absorption spectra, and (C) CD spectra of Cu(II)-Ac-GACPNRMDAAAAAAAADHIMD-NH_2_; metal/ligand ratio 1:1; *C*_L_ = 5 × 10^–4^ M. For clarity, the charges on the speciation plot were omitted (see Table 2A).

In the case of the **Cu(II)-Ac-GACPNRMDAAAAAAAADHIMD-NH**_**2**_ OprC fragment complex, six mononuclear species exist in the studied pH values (Figure 1A). The first formed species is [CuH_2_L], in which three carboxylic groups from aspartic acid are deprotonated and can participate in Cu(II) binding at low pH. The next form, [CuHL]^−^, results from the deprotonation of a His imidazole nitrogen. The lower p*K*_a_ value compared to free ligand provide 1N_im_ binding mode suggesting that the residue is involved in Cu(II) binding (Table 2A). Moreover, the d–d band at 715 nm with ε = 13.0 M^–1^ cm^–1^ (Figure 1B and Table 2A) and EPR parameters *A*_II_ = 150 G and *g*_II_ = 2.33 (Figure S2A and Table 2A) strongly support the presence of 1 nitrogen atom in the coordination sphere.^23,27,28^ The CuL species, with maximum concentration at pH 7, results from the deprotonation and Cu(II) binding to the first amide nitrogen from the peptide backbone. The 2N binding for [CuL]^2–^ species is confirmed by a d–d band at 633 nm with ε = 48.5 M^–1^ cm^–1^ (Figure 1B and Table 2A).^29^ The [CuH_–1_L]^3–^ species, with a maximum concentration at pH 8.5, may result from the deprotonation and Cu(II) binding to the Cys thiol group. The presence of a band at around 330 nm may prove S_Cys_-Cu(II) charge transfer and involvement of the cysteine sulfur in binding (Figure 1B,C).^30,31^ The lowered cysteine p*K*_a_ value in the complex in relation to the free ligand (p*K*_a_ 9.56 (ligand) → p*K*_a_ 7.49 (complex)) strongly suggests that the cysteine residue is involved in the Cu(II) ion coordination. Additionally, on the CD spectra, two positive bands at 537 and 647 nm indicate the Cu(II)-amide binding with a slight tendency to form square-planar geometry with a 1N_im_, 1N^–^, and 1S^–^ binding mode (Figure 1C). The involvement of two nitrogen atoms in Cu(II) binding is confirmed by UV–vis (d–d band at 603 nm with ε = 58.4 M^–1^ cm^–1^) and EPR parameters (*A*_II_ = 160 G and *g*_II_ = 2.24) (Figures 1B, S2A and Table 2A). The loss of the next proton leads to the [CuH_–2_L]^4–^ complex species, with a maximum concentration at pH 10. At this pH, a hypsochromic effect is observed in the UV–vis spectra, from 603 to 570 nm, and EPR parameters (*A*_II_ = 170 G and *g*_II_ = 2.22) suggest a 3N coordination mode with a 1N_im_, 2N^–^, and 1S^–^ donor set (Table 2A, Figures 1B and S2A). The presence of a typical negative (λ = 502 nm, Δε = −0.57 cm^–1^ M^–1^) and positive (λ = 646 nm, Δε = 0.56 cm^–1^ M^–1^) Cotton effect for the last [CuH_–3_L]^5–^ species suggests the formation of a square-planar complex (Figure 1C and Table 2A).^32,33^ The involvement of four nitrogen atoms 1N_im_, 3N^–^ in the binding is confirmed by the blue shift of the d–d band from 570 to 521 nm in UV–vis spectra (Figure 1B and Table 2A).^34,35^ To this species, the 1N_im_, 3N^–^ binding mode was assigned. At pH 10.5–11, a flattening of the band at 330 nm was also observed (Figure 1B), suggesting displacement of the cysteinyl residue by the coordinating third amide.

**Table 2:** Stability Constants, Spectroscopic Parameters, and Proposed Coordination Modes for Cu(II) Complexes with (A) Ac-GACPNRMDAAAAAAAADHIMD-NH_2_, (B) Ac-EMMTPHHQDAIDMAEMALQKAEHPE-NH_2_, and (C) Ac-GMMGMHQGHGMMAMD-NH_2_ at *T* = 298 K^a^

				UV				CD		EPR		
	log β*_ijk_*^b^	p*K*_a_^c^		λ [nm]		ε [cm^–1^ M^–1^]		λ [nm]	Δε [cm^–1^ M^–1^]	*A*_II_	*g*_II_	donors
(A) Cu(II)-Ac-GACPNRMDAAAAAAAADHIMD-NH_2_												
[CuH_2_L]	19.71(6)											
[CuHL]^−^	14.66(4)	5.05		715		13.0				150	2.33	1N_im_
[CuL]^2–^	8.22(4)	6.44		633		48.5		253	1.76	160	2.28	1N_im_, 1N^–^
								340	–0.11			
								545	0.12			
								650	0.19			
[CuH_–1_L]^3–^	0.73(5)	7.49		603		58.4		249	4.55	160	2.24	1N_im_, 1N^–^, 1S_Cys_^–^
				330		537		342	–0.19			
								537	0.29			
								647	0.41			
[CuH_–2_L]^4–^	–8.65(7)	9.38		570		169		249	4.24	170	2.22	1N_im_, 2N^–^, 1S_Cys_^–^
				330		704		343	–0.14			
								543	0.15			
								649	0.43			
[CuH_–3_L]^5–^	–19.72(9)	11.07		521		194		258	4.15	185	2.20	1N_im_, 3N^–^
								300	–0.20			
								321	0.27			
								502	–0.57			
								646	0.56			
(B) Cu(II)-Ac-EMMTPHHQDAIDMAEMALQKAEHPE-NH_2_												
[CuH_5_L]^+^	41.74(3)									135	2.36	
[CuH_3_L]^−^	32.82(3)			719		46.7						1N_im_
[CuH_2_L]^2–^	27.80(7)	5.02		687		56.4				170	2.29	2N_im_
[CuHL]^3–^	22.65(5)	5.15		678		67.9						2N_im_
[CuL]^4–^	17.02(4)	5.63		647		79.7				180	2.27	2N_im_
[CuH_–1_L]^5–^	10.29(5)	6.73		615		92.5		261	1.18	185	2.25	2N_im_, 1N^–^
								534	0.33			
[CuH_–2_L]^6–^	2.51(5)	7.78		593		110		263	3.36	185	2.25	1N_im_, 2N^–^
								534	0.41			
[CuH_–3_L]^7–^	–5.72(5)	8.23		512		122		264	3.79	195	2.23	1N_im_, 3N^–^
								323	0.25			
								477	–0.52			
								622	0.59			
[CuH_–4_L]^8–^	–15.28(5)	9.56		509		123		264	3.66	200	2.23	1N_im_, 3N^–^
								320	0.54			
								488	–1.03			
								618	0.96			
(C) Cu(II)-Ac-GMMGMHQGHGMMAMD-NH_2_												
[CuH_2_L]^3+^	16.76(5)											
[CuHL]^2+^	12.32(4)		4.44		701		82.1	253	0.27			1N_im_
[CuL]^+^	7.15(4)		5.17		639		119	252	1.28			2N_im_
[CuH_–1_L]	0.40(5)		6.75		574		160	247	2.76			2N_im_, 1N^–^
								314	–0.66			
								518	0.56			
[CuH_–2_L]^−^	–6.37(5)		6.77		554		173	242	2.87			2N_im_, 2N^–^
								273	1.78			
								312	–1.17			
								515	0.95			
								605	–0.18			
[CuH_–3_L]^2–^	–14.41(5)		8.04		532		191	230	3.35			1N_im_, 3N^–^
								275	1.11			
								312	–1.24			
								512	0.96			
								600	–0.46			

a Values in parentheses are standard deviations on the last significant figure.

b Cu(II) stability constants are presented as cumulative log β*_ijk_* values. *L* stands for a fully deprotonated peptide ligand that binds Cu(II) ions: β(*M_i_H_j_L_k_*) = [*M_i_H_j_L_k_*]/([*M*]*_i_*[*H*]*_j_*[*L*]*_k_*), where [*L*] is the concentration of the fully deprotonated peptide.

c p*K*_a_ = log β (*M_i_H_j_* + 1*L_k_)* – log β(*M_i_H_j_L_k_*).

It is worth noticing that all analyzed peptide domains also contain methionyl residues in their sequences. Due to the lack of deprotonating side chains in the methionyl residue (–SCH_3_ group), the participation of thioether groups in the binding of Cu(II) ions cannot be confirmed or excluded using potentiometric titrations. Also, UV–vis spectroscopy will not tell us more about Met–Cu(II) interactions. The existence of a CT band on UV–vis spectra around 320–330 nm was assigned to S(thioether) → Cu(II) interactions in the literature, but this was analyzed for tripeptides MGG, GMG, and GGM only.^36^ For our systems, where the bands in the range 200–370 nm can be assigned to *n* → π*/π → π* intraligand transitions associated to other amino acid present in the sequences,^37^ specific S(Met) → Cu(II) bands are very likely to be overlapped. Moreover, in this UV region, strong *n* → π* and π → π* peptide backbone transitions are also present.^38^ Thus, the influence of thioether groups of methionyl in the Cu(II) coordination had to be confirmed by using NMR spectroscopy.

**Figure 2:**
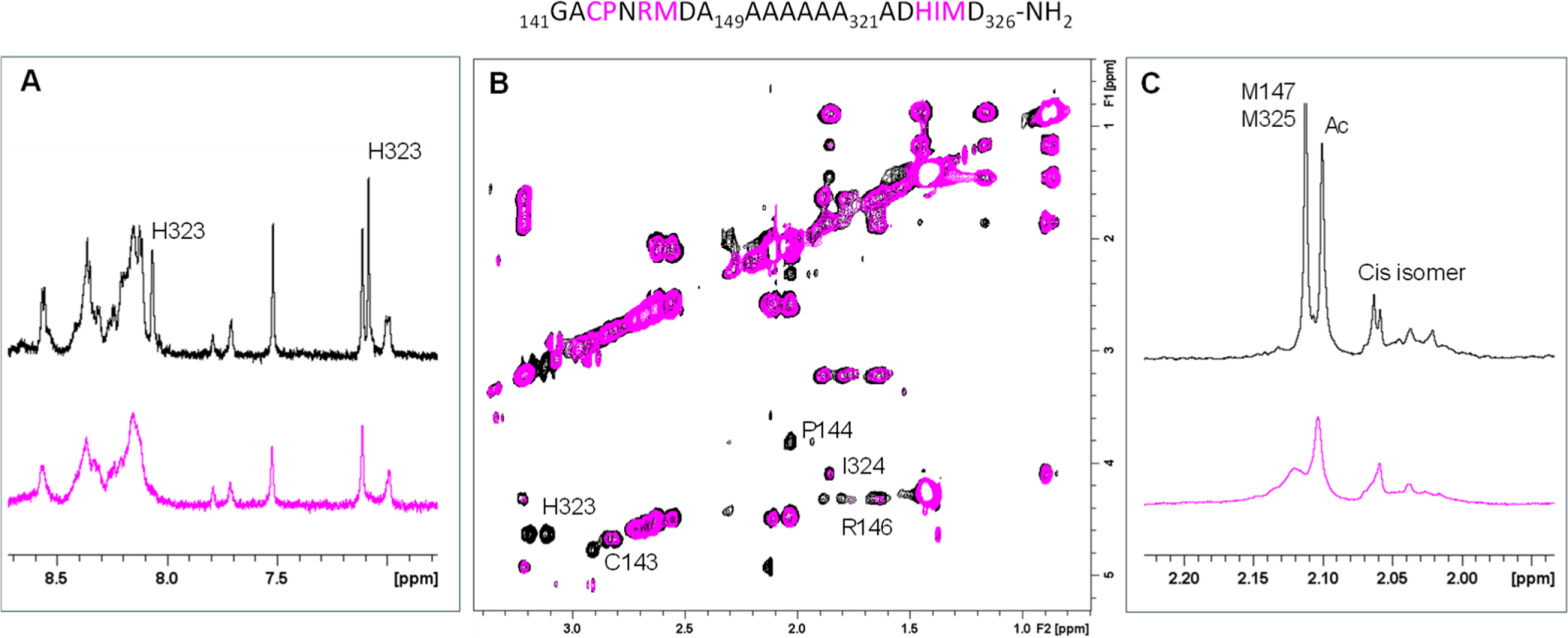
Superimposition of NMR spectra of Ac-_141_GACPNRMDA_149_(AAAAAA)_321_ADHIMD_326_-NH_2_ (0.5 mM) in the absence (black) and presence (magenta) of 0.2 Cu(II) equivalents. (A) 1D ^1^H amide and aromatic region, (B) 2D ^1^H–^1^H TOCSY aliphatic region, and (C) 1D ^1^H aliphatic region. Spectra were recorded at 288 K in 20 mM phosphate buffer, pH 7.4. The peptide sequence is shown at the top with the most affected residues highlighted in magenta.

Cu(II) to the **Ac-**_**141**_**GACPNRMDA**_**149**_**(AAAAAA)**_**321**_**ADHIMD**_**326**_**-NH**_**2**_ OprC system was investigated using NMR spectroscopy. NMR spectra of the peptide, both in the presence and absence of the metal ion, were recorded at 288 K to monitor the signals of NH protons, which appeared very broad at room temperature, as expected for disordered peptides. All NMR spectra were acquired for peptide solutions in phosphate buffer (pH 7.4) to avoid any shifts due to subtle pH changes. The addition of substoichiometric amounts of Cu(II) (0.2 eqs.) led to selective line broadening of NMR resonances belonging to residues near the metal center. This broadening arises from dipolar coupling between the copper’s unpaired electron and the nuclei in close proximity to the metal-binding site.^39−42^Figure 2 shows selected regions of the one-dimensional (1D) and 2D NMR spectra, highlighting the most affected amino acids, including Cys143, His323, and nearby residues, such as Pro144 and Ile324. Additionally, the methyl protons of both methionine residues are significantly impacted by the presence of the paramagnetic ion. The proximity of Met145 to the copper coordination sphere is further supported by the effects observed on Arg146, whose scalar correlations, visible in the TOCSY spectrum, completely disappeared upon the addition of the copper ion. Finally, the analysis of the fingerprint region of the TOCSY spectrum confirmed the previously observed effects on His323 and Ile324, along with a slight variation on Met325 (data not shown). The data indicate copper binding to Cys143 and His323, with the possible involvement of the Met-SCH_3_ groups in the metal coordination sphere.

**Figure 3:**
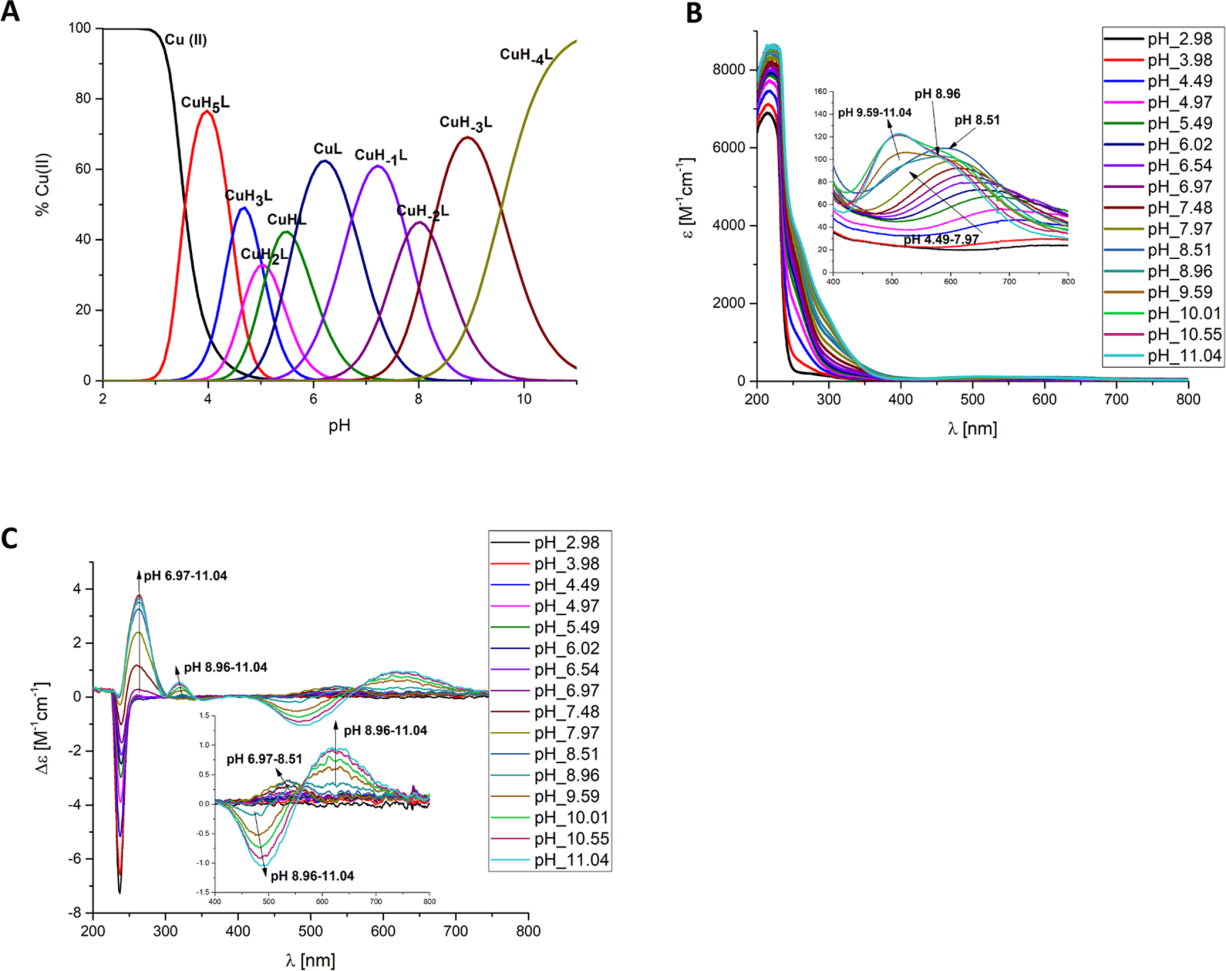
(A) Species distribution diagram, (B) UV–vis absorption spectra, and (C) CD spectra of Cu(II)-Ac-EMMTPHHQDAIDMAEMALQKAEHPE-NH_2_; metal/ligand ratio 1:1; *C*_L_ = 5 × 10^–4^ M. For clarity, the charges on the speciation plot were omitted (see Table 2B).

In the Cu(II) binding site of the bacterial metallophore CopM with a characteristic MxxHH motif, **Ac-EMMTPHHQDAIDMAEMALQKAEHPE-NH**_**2**_, nine Cu(II) complexes were detected (Figure 3A and Table 2B). The first complex detected by potentiometry is [CuH_5_L]^+^ with a maximum at around pH 3.9. The stoichiometry of this species indicates that five acid–base sites are already deprotonated: three Glu and two Asp residues have lost a proton. At this point, most probably, Cu(II) is anchored by carboxyl groups of acidic amino acids.^43−45^ The first spectroscopically detectable species is [CuH_3_L]^−^, with a maximal concentration at pH 4.7 where one histidine imidazole should be bound to the copper(II) ion (λ = 719 nm, ε = 46.7 cm^–1^ M^–1^) (Figure 3B and Table 2B). The next deprotonation step (p*K*_a_ = 5.02) for the [CuH_2_L]^2–^ species led to the formation of a 2N_im_ complex, as indicated by spectroscopic UV–vis and EPR parameters (λ = 687 nm, ε = 56.4 cm^–1^ M^–1^, *g*_II_ = 2. 29, *A*_II_ = 170) (Figures 2B and S2B). For the next two species, [CuHL]^3–^ and [CuL]^4–^, no significant shifts in the UV–vis spectra are observed, indicating no changes in the 2N binding mode. The calculated p*K*_a_ values, 5.15 for [CuHL]^3–^, could result from the deprotonation of an unbound histidine residue (Table 2B). The observed decrease of the p*K*_a_ value for the third histidine (5.15) in the [CuHL]^3–^ species compared to the free ligand [H_2_L]^4–^ (7.43) suggests that Cu(II) can coordinate to all imidazoles present in the sequence but only to two at the same time. The p*K*_a_ value (5.63) for [CuL]^4–^ species cannot be assigned to the deprotonation and coordination of the first amide donor (any LMCT N^–^ → Cu(II) bands on CD spectra at pH 6 where [CuL]^4–^ predominates), but at the same time, this p*K*_a_ value is rather too low to indicate coordination of the water molecule.^29,46,47^ This may suggest that, in the [CuL]^4–^ complex, copper is bound to the two imidazole nitrogen and carboxyl groups of aspartic acid. The involvement of aspartic acid in binding or stabilizing the Cu(II) binding site is further confirmed by NMR studies recorded at pH 7.4 (see the description below). A similar phenomenon was observed in a recently published paper, where the coordination properties of Cu(II) ions toward the peptides Ac-GSTENLKH-NH2 (R1) and Ac-GS(P)TENLKH-NH2 (R1P) (Ser phosphorylated analogue) were studied.^48^ The next three species, [CuH_–1_L]^5–^, [CuH_–2_L]^6–^, and [CuH_–3_L]^7–^, result from the copper interaction with the amide group of the peptide chain, forming 3N and 4N complexes. The involvement of an increasing number of nitrogen atoms in Cu(II) binding is proven by a blue shift, 647 → 511 nm, in the UV–vis spectra (Figure 3B and Table 2B). The square-planar 1N_im_, 3N^–^ complex appeared at pH 9, as indicated by the typical shape of CD bands in the visible region (positive and negative Cotton effect at λ = 622 nm, Δε = 0.59 cm^–1^ M^–1^ and λ = 477 nm, Δε = −0.52 cm^–1^ M^–1^, respectively) (Figure 3C and Table 2B). The last [CuH_–4_L]^8–^ species with p*K*_a_ = 9.56 results from the deprotonation of a lysine residue, which does not participate in the binding (p*K*_a_ = 9.94 for the free ligand) (Table 2B).

**Figure 4:**
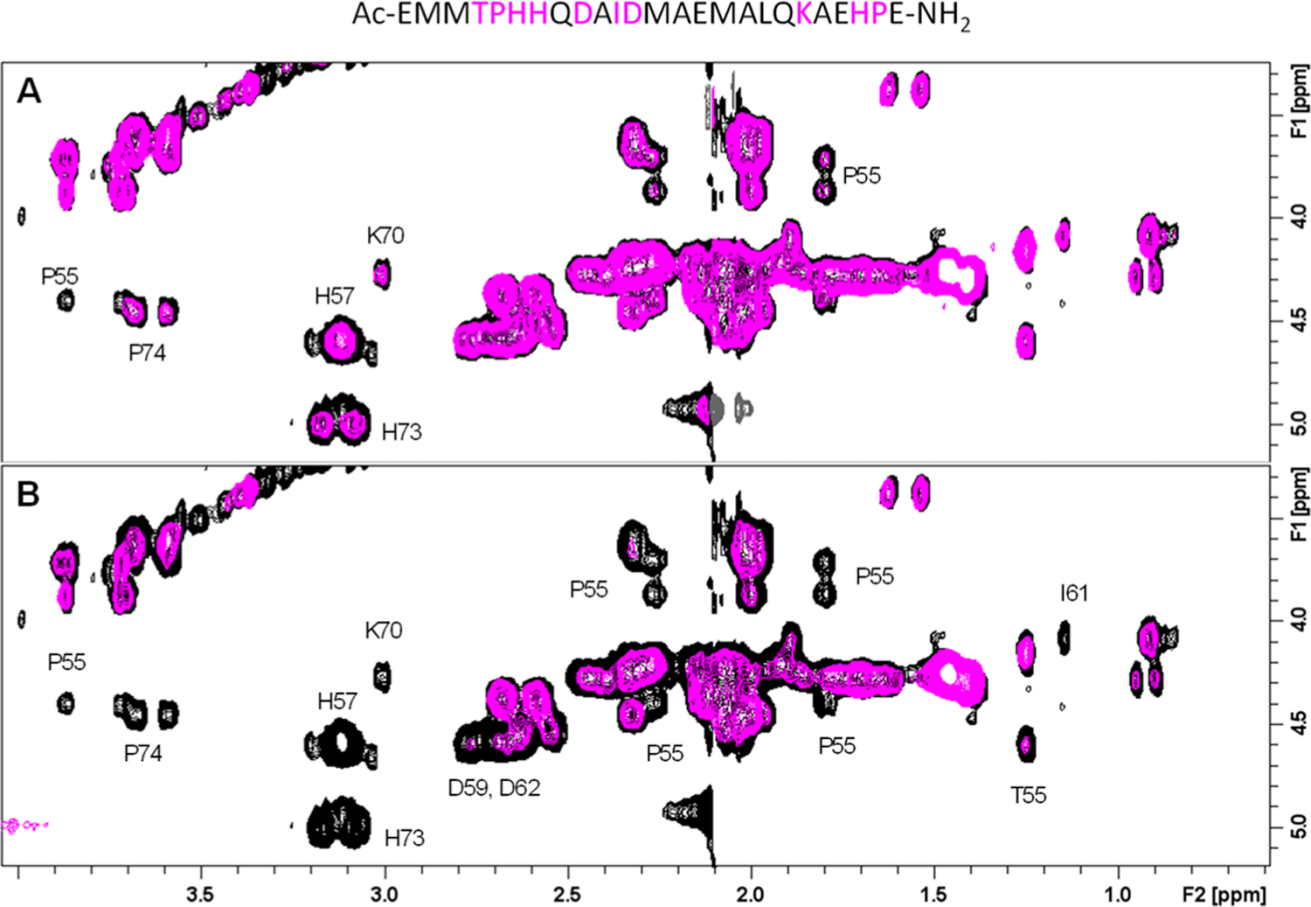
Superimposition of the aliphatic regions of 2D ^1^H–^1^H TOCSY NMR spectra of Ac-_51_EMMTPHHQDAIDMAEMALQKAEHPE_75_-NH_2_ (0.5 mM) in the absence (black) and presence (magenta) of (A) 0.1 and (B) 0.2 Cu(II) equivalents. Spectra were recorded at 288 K in 20 mM phosphate buffer, pH 7.4. The peptide sequence is shown at the top, with the most affected residues highlighted in magenta.

NMR analysis indicated that, after the addition of 0.1 equiv of copper to **Ac-**_**51**_**EMMTPHHQDAIDMAEMALQKAEHPE**_**75**_**-NH**_**2**_, the most affected cross-peaks were those of the histidines, prolines, and Lys70, as shown in the TOCSY spectrum (Figure 4A). The subsequent addition of 0.2 equiv of Cu(II) not only intensified the effects on the previously mentioned residues but also caused further broadening of the correlations associated with Thr55, Asp59, Ile61, and Asp69 (Figure 4B). At the same time, analysis of the 1D spectrum revealed not only the effects on the aromatic proton of the three histidines but also the selective broadening of the methyl groups of methionines (Figure S3). Notably, the singlets corresponding to the –SCH_3_ groups were affected differently, with two of the four observed signals being more influenced than the others. These results support copper coordination to the three histidines through their imidazole nitrogens (the spectroscopic parameters indicate binding to two His residues at the same time and possible fast exchange with the third residue) and suggest the involvement of the carboxyl group of one of the two aspartates in completing the coordination sphere. The involvement of the carboxyl group in the binding of Cu(II) ions in the studied peptide seems to be entirely possible in relation to the literature data describing the binding of Cu(II) ions to two MxxHH motifs of the CopM transporter, where, in addition to two histidine residues (one from each motif), Asp59 also participates in the binding.^15^

**Figure 5:**
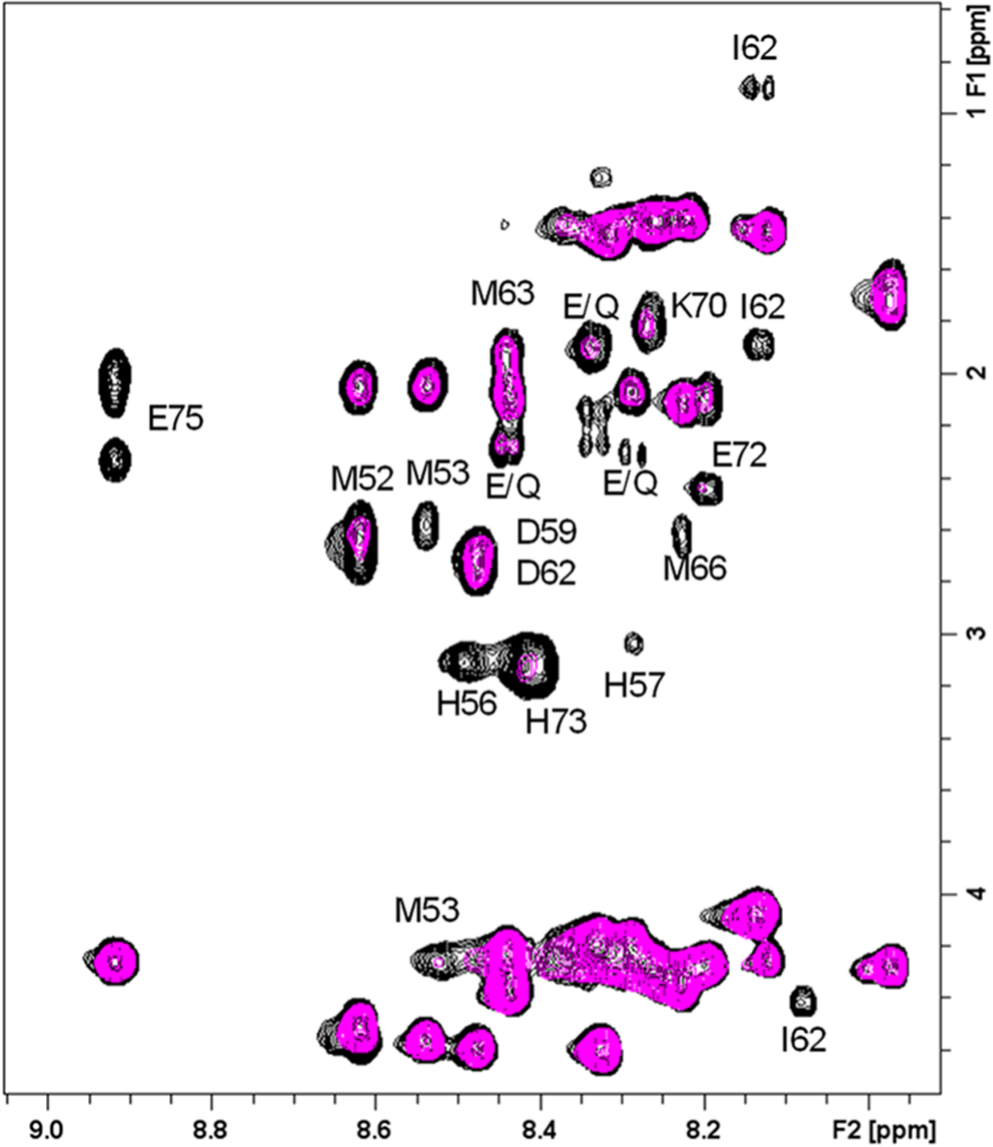
Superimposition of the fingerprint regions of 2D ^1^H–^1^H TOCSY NMR spectra of Ac-_51_EMMTPHHQDAIDMAEMALQKAEHPE_75_-NH_2_ (0.5 mM) in the absence (black) and presence (magenta) of 0.1 Cu(II) equivalents. Spectra were recorded at 288 K in 20 mM phosphate buffer, pH 7.4. The label E/Q refers to correlations that were not unequivocally assigned to Glu or Gln residues.

To better understand which methionine residues are most affected by the paramagnetic ion and to have a better characterization of the metal coordination sphere, we analyzed the effect of copper on the amide proton correlations by comparing the fingerprint regions of the TOCSY spectrum in the absence and presence of Cu(II) (Figure 5). The data indicate that even after the addition of 0.1 equiv of copper, substantial changes are observed in many of the correlations. In particular, beyond the previously noted effects, broadening is seen in other residues primarily located in the N-terminal (Met52 and Met53) and C-terminal regions (Met66, Glu72, and Glu75). Further addition of 0.2 equiv results in excessive broadening, preventing the extraction of detailed information. Among the methionine residues, the most pronounced effects are observed on Met53 and Met66, both likely being involved in the stabilization of the binding site. In the first case, Met53 may be facilitated by its proximity to the His–His pair. In the second case, Met66 could be located near the binding site due to backbone folding induced by His73 coordinating with the paramagnetic ion.

**Figure 6:**
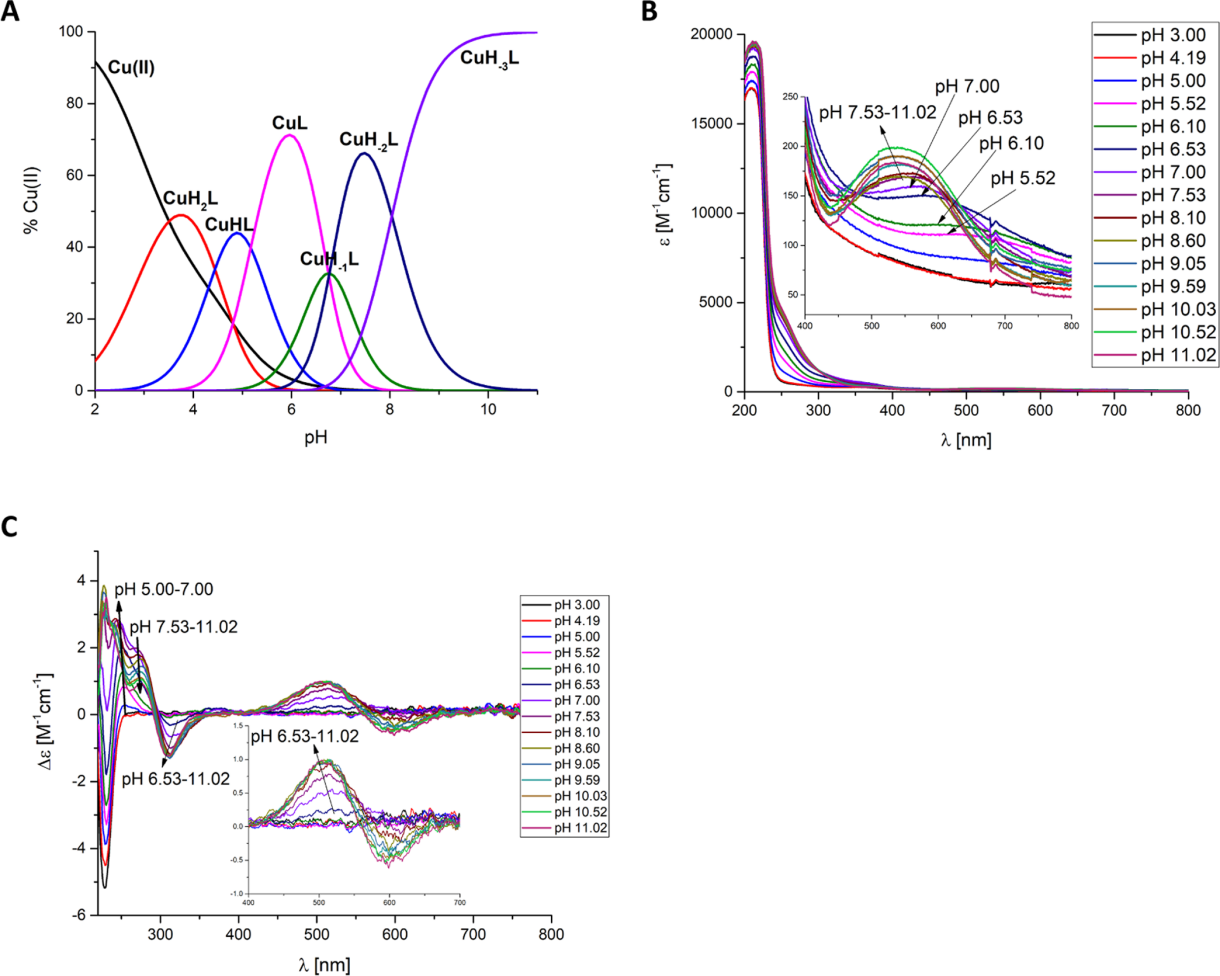
(A) Species distribution diagram, (B) UV–vis absorption spectra, and (C) CD spectra of Cu(II)-Ac-GMMG**MH**QG**H**GMMAMD-NH_2_; metal/ligand ratio 1:1; *C*_L_ = 5 × 10^–4^ M. For clarity, the charges on the speciation plot were omitted (see Table 2C).

In the case of the Cu(II) binding site in the CopM metallophore, **Ac-GMMGMHQGHGMMAMD-NH**_**2**_ (which contains the MHxxH motif), Cu(II) begins to coordinate to this ligand at a very low pH (Figure 6A). The first detected complex is [CuH_2_L]^3+^, where the carboxyl group of the Asp residue present in the peptide sequence may participate in the coordination at acidic pH. Upon an increase in pH, the two histidines with p*K*_a_ values of 4.44 and 5.17 undergo deprotonation, leading to the formation of the [CuHL]^2+^ and [CuL]^+^ species (Table 2C and Figure 6B). The involvement of imidazole nitrogens of two histidines in Cu(II) binding provides a characteristic charge transfer N_im_-Cu(II) band present at around 250 nm on the CD spectra (Figure 6C). Moving to alkaline conditions, the CD spectra show an increasing signal in the visible region, which can be ascribed to the backbone amides. The [CuH_–1_L] species, which reaches its maximum concentration at pH 6.80, comes from deprotonation of the amide nitrogen. In the UV–vis spectrum, the maximum absorption at 574 nm is observed, which supports a three nitrogen 2N_im_, 1N^–^ binding mode in the coordination sphere of Cu(II) (Figure 6B and Table 2C). Above pH 7.50, where the [CuH_–2_L]^−^ complex dominates, a UV–vis band with a maximum absorption at 554 nm indicates the formation of a 4N complex (Figure 6B and Table 2C). Additionally, the CD spectrum shows positive and negative Cotton effects at 515 and 605 nm, respectively. These observations indicate the typical square-planar geometry associated with the compound (Figure 6C). The last species, [CuH_–3_L]^2–^, results from the replacement of one imidazole nitrogen atom by an amide, indicating a 1N_im_, 3N^–^ set of donors (Table 2C).

**Figure 7:**
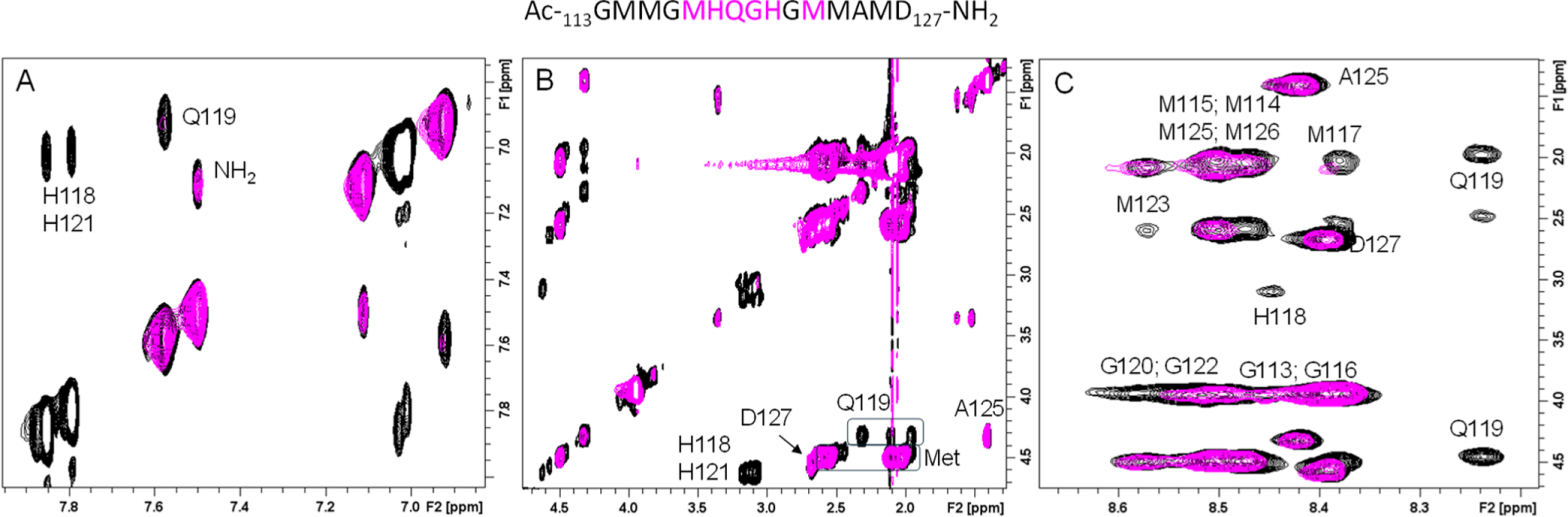
Superimposition of (A) aromatic, (B) aliphatic, and (C) fingerprint regions of 2D ^1^H–^1^H TOCSY NMR spectra of Ac-_113_GMMGMHQGHGMMAMD_127_-NH_2_ (0.5 mM) in the absence (black) and presence (magenta) of 0.1 Cu(II) equivalents. Spectra were recorded at 288 K in 20 mM phosphate buffer, pH 7.4. The peptide sequence is shown at the top, with the most affected residues highlighted in magenta.

The NMR analysis of **Ac-**_**113**_**GMMGMHQGHGMMAMD**_**127**_**-NH**_**2**_ was conducted in the same manner as that for the two previous systems. Following the addition of copper, which caused selective variations in the NMR signals, the regions of the 1D and 2D spectra most affected by the paramagnetic ion were identified. The 1D spectrum once again revealed broadening of the aromatic signals of the histidines along with selective broadening of some thioether group signals from the six methionine residues (Figure S4). At the same time, the analysis of the 2D ^1^H–^1^H TOCSY maps confirmed the effects on His118 and H121 highlighting the broadening of Gln119 and Gly120, located between the histidine residues, along with variations in the cross-peaks of Met117 and Met123 (Figure 7). These findings strongly suggest Cu(II) binding to both the imidazole nitrogens of the histidine and the thioether groups of Met117 and Met123.

**Figure 8:**
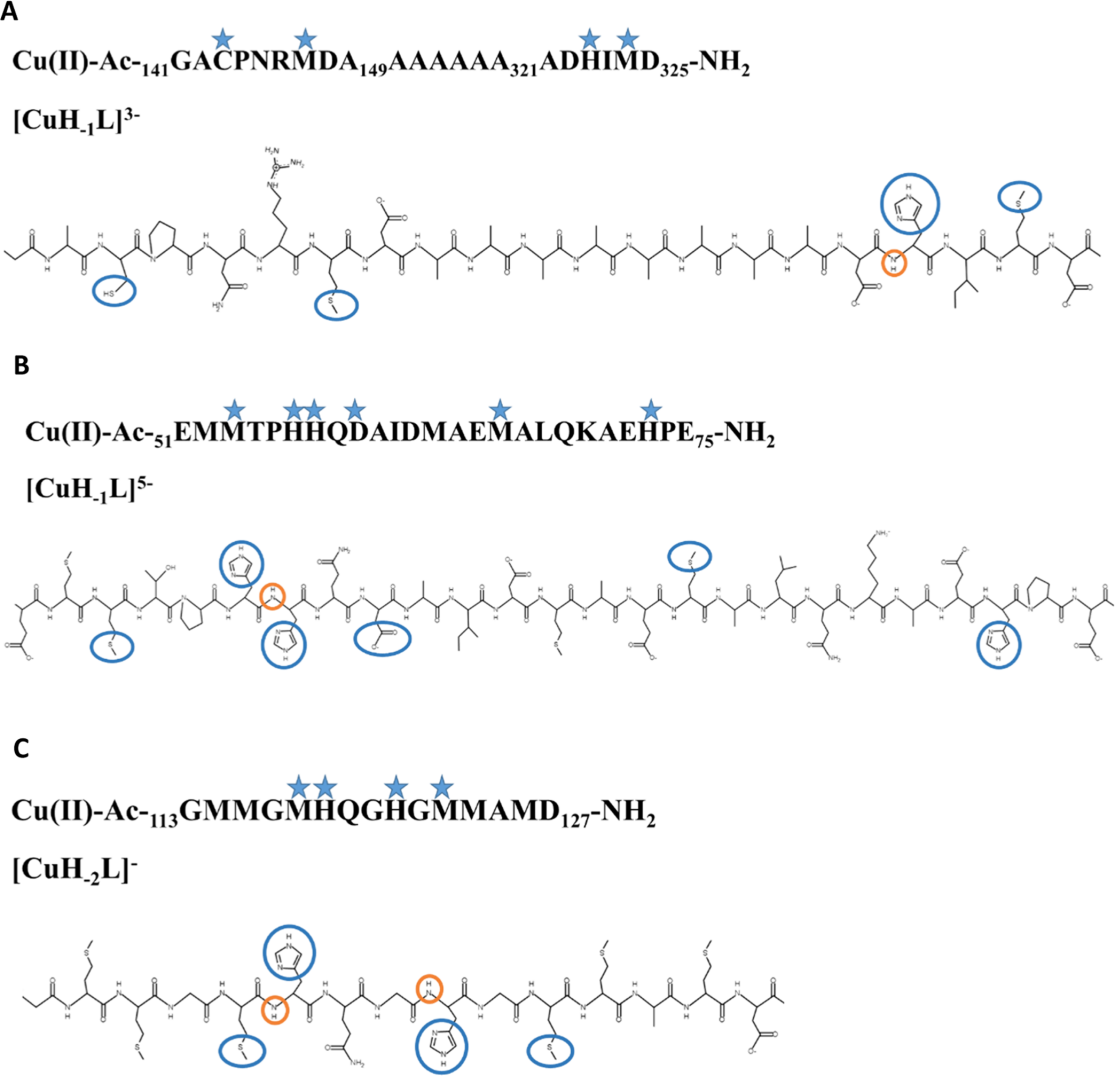
Scheme showing the most probable donor atoms involved in the coordination of Cu(II) ions in (A) OprC CxxxM-HxM domain, (B) CopM MxxHH domain, and (C) CopM MHxxH domain. Side groups of binding amino acids are highlighted with blue circles and potential amide nitrogens of the peptide bond completing the coordination sphere are highlighted with orange circles.

Taking into account the potentiometric and spectroscopic (UV–vis, CD, EPR, and NMR) studies, the most probable donor groups of the OprC and CopM complex species dominating at pH 7.4 are presented in Figure 8.

### Can Cu(II) Coordination Affect the Structures of OprC and CopM Domains?

In order to have very basic information about structural properties of investigated ligands and their Cu(II) complexes, far-UV CD spectroscopy was used to monitor the structural changes after adding metal in the pH range 3–11 (Figure S5). Although the addition of the copper ions does not change the structure of the investigated ligands, the CD spectra for the apo forms, in particular, OprC and CopM MxxHH metal-binding domains, seem very interesting (Figure S5A,B). At acidic pH, both ligands adopt an α helical structure. In the case of Ac-EM**M**TP**HH**QDAIDMAEMALQKAEHPE-NH_2_, the clearly outlined two negative absorption bands at 205 and 225 nm (Figure S5B) indicate an even higher percentage of the α helical structure than in the case of the OprC domain (Figure S5A). For both ligands, as the pH value increases, the spectrum shows a shift toward shorter wavelengths (205 → 200 nm, hypsochromic effect) and a decrease in the intensity of the negative band at around 225 nm. This indicates a tendency for the structure to change from α-helical to disordered in increasing pH values. The change in the structure with increasing pH is probably related to the change in the charge of the peptides, indicating the deprotonation of the side chains of amino acids present in the sequences (Asp, Glu, His, Cys, and Lys). The CopM MHxxH domain and its Cu(II) complexes on CD spectra show a strong negative absorption band at 197 nm, confirming a random coil structure in all pH ranges regardless of the presence or absence of Cu(II) (Figure S5C).

### Comparison of the Thermodynamic Stability of an Unprecedented CxxxM-HxM Metal-Binding Site of OprC with Two MxxHH and MHxxH Motifs of CopM

**Figure 9:**
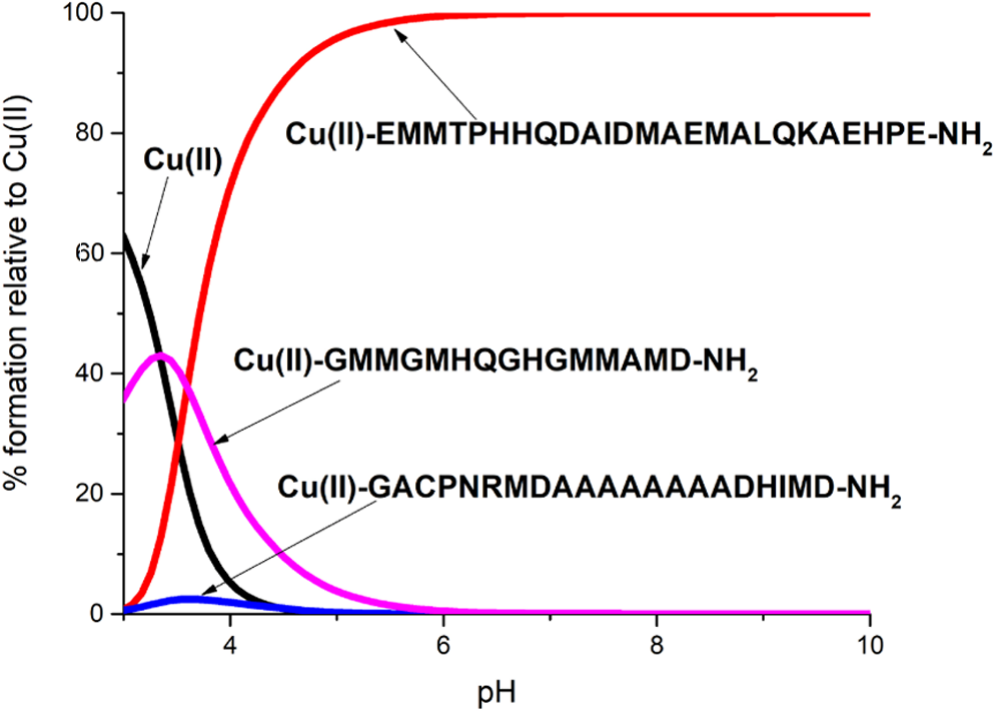
Competition plots for the Cu(II) complexes with Ac-GACPNRMDAAAAAAAADHIMD-NH_2_ (CxxxM-HxM metal-binding site of OprC), Ac-EMMTPHHQDAIDMAEMALQKAEHPE-NH_2_ (CopM MxxHH metal-binding domain), and Ac-GMMGMHQGHGMMAMD-NH_2_ (CopM MHxxH metal-binding domain) based on potentiometric data (Tables 1 and 2).

There is a significant difference in the efficiency of metal-binding between the OprC and CopM domains, as visualized on the competition plot, based on the potentiometric stability constants, which simulates a hypothetical situation in which equal amounts of Cu(II) and the studied peptides are present in solution. Above pH 4, the CopM MxxHH metal-binding domain is a much more efficient Cu(II) binder than its MHxxH metal-binding domain and CxxxM-HxM metal-binding site of OprC (Figure 9). At pH 7.4, which is the most interesting (physiological) value in most biological systems, almost 100% of the available Cu(II) would be bound to Ac-EM**M**TP**HH**QDAIDMAEMALQKAEHPE-NH_2_ (CopM MxxHH metal-binding domain). In our hypothetical simulation, the CopM MHxxH metal-binding domain and CxxxM-HxM metal-binding site of the transmembrane OprC have no chance in competition with the Ac-EM**M**TP**HH**QDAIDMAEMALQKAEHPE-NH_2_ fragment. It seems that the CopM MxxHH motif, where two coordinating histidine residues are localized next to each other, is much more effective in Cu(II) binding than the CopM MHxxH motif where two binding histidines are separated by two other amino acid residues. Moreover, the CxxxM-HxM metal-binding site of OprC, where, in addition to histidine, cysteine residue may also bind Cu(II) ions, does not constitute any competition in Cu(II) binding for the CopM MxxHH domain. The obtained comparison may suggest that the CopM metallophore (more precisely, its MxxHH binding site) binds Cu(II) ions very strongly and is unable to give them back to the OprC transmembrane transporter; therefore, it is perhaps transported together with copper ions through OprC into the bacterial cell.

**Figure 10:**
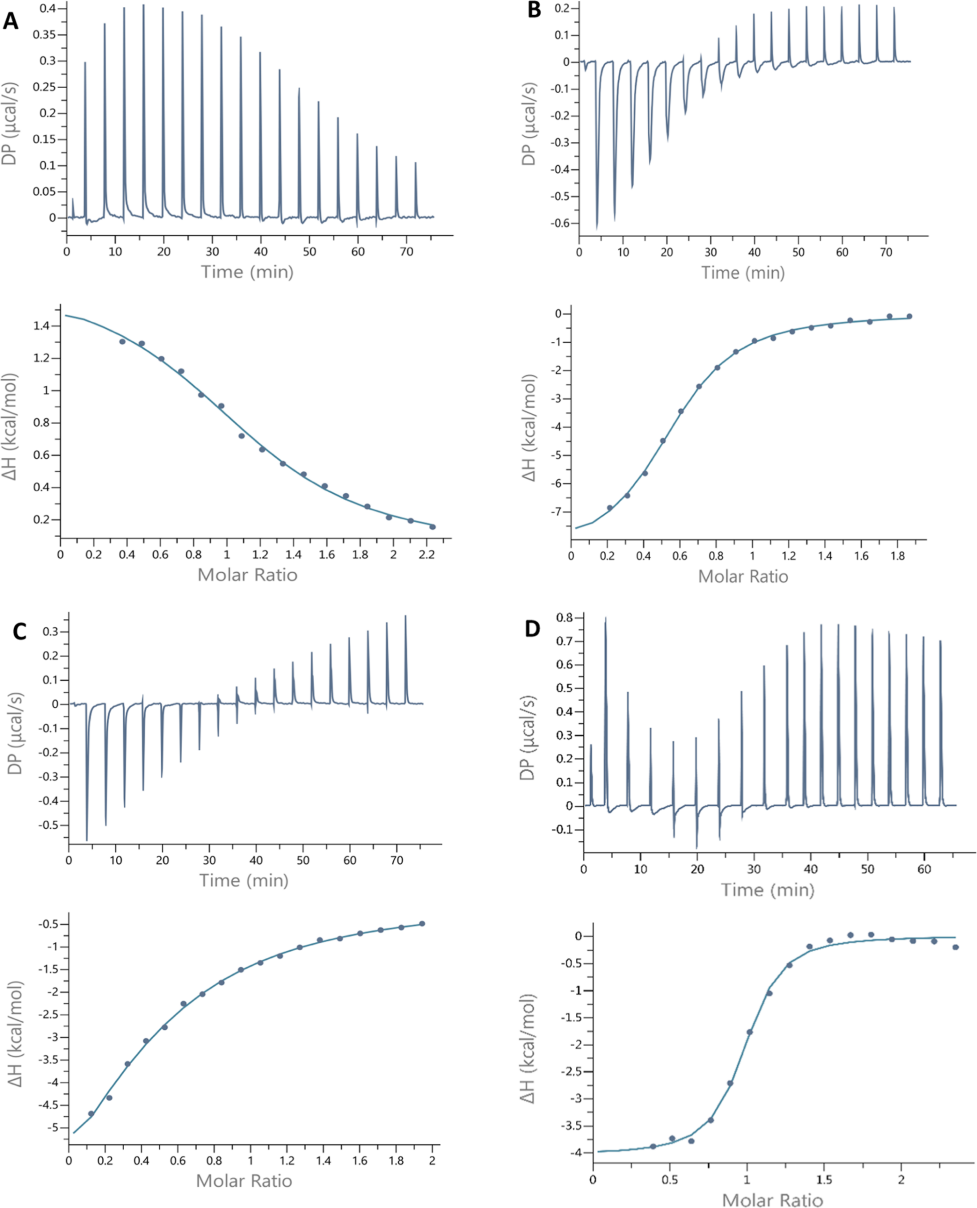
Representative ITC data (above) and their corresponding thermodynamic signatures (below) for Cu(II) (1.0–2.0 mM) titrated into (A) Ac-GACPNRMDAAAAAAAADHIMD-NH_2_, (B) Ac-EMMTPHHQDAIDMAEMALQKAEHPE-NH_2_, and (C) Ac-GMMGMHQGHGMMAMD-NH_2_ peptides (100–200 μM) at pH 7.0. (D) Representative ITC data for the reverse titration (1 mM Ac-EMMTPHHQDAIDMAEMALQKAEHPE-NH_2_ peptide to 100 μM Cu(II)), pH 7.0.

To better understand the interaction between OprC and CopM protein (in particular, its CopM MxxHH domain, which forms the most stable Cu(II) complexes; see Figure 9), the ITC measurements were a perfect method of choice. ITC provides conditional results, which perfectly compare the findings for all three peptides, as the measurements were performed under precisely the same conditions. The best-fit values for stoichiometry (*n*_ITC_), stability constant (*K*_d ITC_), and change in enthalpy (Δ*H*_ITC_) were determined by using a one-site binding model. All three peptides exhibit moderate affinities for Cu(II) binding, with the Ac-EM**M**TP**HH**QDAIDMAEMALQKAEHPE-NH_2_ peptide displaying the highest affinity, which is consistent with our potentiometric results. The OprC Ac-GA**C**PNR**M**DAAAAAAAAD**H**I**M**D-NH_2_ peptide’s binding to Cu(II) is primarily driven by a net favorable change in entropy. Both CopM binding sites, the Ac-EM**M**TP**HH**QDAIDMAEMALQKAEHPE-NH_2_ and Ac-GMMG**MH**QG**H**GMMAMD-NH_2_ peptides, exhibit a biphasic binding pattern to Cu(II) ions at this pH (Figure 10). In contrast to the CxxxM-HxM binding domain of OprC, these two peptides may exhibit an M/L 1:2 stoichiometry, indicating that two CopM domains can form bis-complexes. This observation aligns with our previous findings involving poly-His and poly-Asp peptides, which were titrated with metal ions at a specific pH.^43,49^ However, we emphasize here that the formation of complexes with stoichiometry other than M/L 1:1 was not confirmed by MS and potentiometric studies. Several factors could explain the formation of M/L 1:2 complexes in ITC. Most probably, when a peptide is titrated into a solution of metal ions, the peptide concentration is much lower, which can lead to a different interaction profile. The saturation of binding sites may occur more slowly in this reversed titration, and the thermodynamics could differ due to the changed molar ratios. The system’s response can vary significantly depending on whether metal ions are the limiting factor (as in the first case) or whether the peptide is at a lower concentration (as in the reverse titration). Additionally, for peptides with multiple charged residues, the peptide may initially interact with the metal in a less ordered manner compared to when metal ions are the limiting reagent and the peptide is more likely to form stable complexes. The way charged residues on the peptide (such as histidines and aspartates) interact with Cu(II) ions can lead to different entropic and enthalpic contributions. Moreover, when titrating the peptide into the metal ion solution, the relative arrangement of charges may change, and the overall complexity of binding could cause entropic contributions to become more significant, which might explain the observed differences in the thermodynamic behavior.

Reversing the titration direction (adding the peptide to the Cu(II) solution) reveals a 1:1 binding for Ac-EM**M**TP**HH**QDAIDMAEMALQKAEHPE-NH_2_ peptide (Figure 10D and Table 3). This data were also fit to a single-site binding model. The reversed titration indicates an even higher affinity of the Ac-EM**M**TP**HH**QDAIDMAEMALQKAEHPE-NH_2_ peptide for Cu(II) (Table 3). To further investigate this, we performed ITC titrations to study the competition for Cu(II) ions between Ac-EM**M**TP**HH**QDAIDMAEMALQKAEHPE-NH_2_ and Ac-GA**C**PNR**M**DAAAAAAAAD**H**I**M**D-NH_2_ peptides. The results showed an affinity of 8.32 ± 0.9 μM when Ac-EM**M**TP**HH**QDAIDMAEMALQKAEHPE-NH_2_ was titrated into Ac-GA**C**PNR**M**DAAAAAAAAD**H**I**M**D-NH_2_ pretreated with Cu(II) in a 1:1 ratio (Figure S6A). In contrast, when Ac-GA**C**PNR**M**DAAAAAAAAD**H**I**M**D-NH_2_ was titrated into Ac-EM**M**TP**HH**QDAIDMAEMALQKAEHPE-NH_2_ pretreated with Cu(II), the DP change was too weak to fit the data to any model and calculate meaningful results (Figure S6B).

**Table 3:** Experimental Thermodynamic Values for Cu(II) Binding to Ac-GACPNRMDAAAAAAAADHIMD-NH_2_ (GAC), Ac-GMMGMHQGHGMMAMD-NH_2_ (GMM), and Ac-EMMTPHHQDAIDMAEMALQKAEHPE-NH_2_ (EMM) from ITC Measurements in 25 mM Caco Buffer, pH 7.0 at 25 °C

	GAC	GMM	EMM	EMM(*r*)
*K*_d ITC_ [μM]	34.0 ± 11.7	97.0 ± 25.00	14.0 ± 1.05	1.48 ± 0.282
Δ*H*_ITC_ [kcal/mol]	1.59 ± 0.20	–11.0 ± 2.49	–8.58 ± 0.199	–4.05 ± 0.11
*n*_ITC_	1.01 ± 0.047	0.452 ± 0.050	0.557 ± 0.006	0.940 ± 0.011
–*T*Δ*S*_ITC_ [kcal/mol]	–7.69	5.54	1.96	–3.91

## Conclusions

In this work, we wanted to find out if it was thermodynamically possible for the CopM metallophore to pass over Cu(II) to the OprC transmembrane protein. In order to find this, we analyzed Cu(II) complexes of a novel **CxxxM-HxM** metal-binding site from the OprC transmembrane protein (Ac-GA**C**PNR**M**DAAAAAAAAD**H**I**M**D-NH_2_) and two metal-binding sites of the putative CopM metallophore (Ac-EM**M**TP**HH**QDAIDMAEMALQKAEHPE-NH_2_ and Ac-GMMG**MH**QG**H**GMMAMD-NH_2_).

All examined complexes exist in equimolar stoichiometry. The spectroscopic and potentiometric studies reveal that the OprC peptide fragment involves Cys143 and His323 in Cu(II) binding, with possible contributions from Met-SCH_3_ groups in the metal coordination sphere. The methyl protons of both methionine residues, located close to Cys and His, are significantly affected by the presence of a paramagnetic ion. In the MxxHH metal-binding domain of CopM (Ac-EM**M**TP**HH**QDAIDMAEMALQKAEHPE-NH_2_), two of the three imidazole groups of histidine residues bind Cu(II); however, both potentiometric and NMR studies indicate a dynamic exchange involving all three histidines present in the sequence. Additionally, broadening of the NMR signals from two methionyl groups (of the four present in the sequence) may suggest the coordination of these groups in an axial position, completing the Cu(II) binding sphere at pH 7. Moreover, NMR studies confirm the involvement of one Asp in Cu(II) binding. In the MHxxH metal-binding domain of CopM (Ac-GMMG**MH**QG**H**GMMAMD-NH_2_), which has a significantly lower affinity for Cu(II) compared to the CopM MxxHH domain, Cu(II) ions bind to two histidyl residues and two of the six methionine thioether groups, located closest to two imidazoles.

The potentiometric and ITC results clearly show a significant difference in the efficiency of metal-binding between the OprC and the CopM domains. At pH 7, the metal-binding site of CopM with the MxxHH motif is the most effective ligand for Cu(II) ions compared to the MHxxH binding site of CopM and the novel CxxxM-HxM metal-binding site from OprC. This finding confirms that two adjacent histidine residues are much more effective in Cu(II) binding than two imidazoles separated by two other amino acid residues. Moreover, the CxxxM-HxM metal-binding site in OprC, where a cysteine residue also binds Cu(II) ions alongside histidine, does not compete effectively for Cu(II) with the MxxHH CopM metal-binding site. This comparison suggests that the MxxHH domain of the CopM metallophore binds Cu(II) ions very strongly and may retain them during transport through OprC, potentially facilitating the passage of both the CopM metallophore and bound copper ions into the bacterial cell.

The obtained results, suggesting a potential interaction between the CopM metallophore fragment and the transmembrane protein OprC in vitro, could initiate highly valuable *in vivo* studies to track and understand the mechanism of copper ion transport by bacteria. Additionally, the potential attachment of an antibiotic to the CopM metallophore fragment, recognized by OprC (Trojan horse strategy), could contribute to the development of new treatment strategies, which is crucial in the era of rapidly advancing antibiotic resistance.

## Supporting Information Available

Mass spectra and *m*/*z* values for Cu(II)-peptide systems; EPR spectra for Cu(II)-peptides; 1H NMR spectra for CopM domains and their Cu(II) complexes; far-UV CD spectra for CopM and OprC domains and its Cu(II) complexes; and ITC titrations to study the competition for Cu(II) ions between CopM and OprC fragments (Figures S1–S6, Table S1) (PDF)
