# Supplementary material for: Thiol, His–His Motif, and the Battle over Cu(II) in the Relationship of CopM Metallophore and OprC Outer Membrane Protein
Source: Inorg Chem. 2025 Feb 6;64(6):2936–50. doi: 10.1021/acs.inorgchem.4c05101 (PMC11836926; doi:10.1021/acs.inorgchem.4c05101)
Supplement: Supplementary file 1 — ic4c05101_si_001.pdf [file ic4c05101_si_001.pdf]

SUPPORTING INFORMATION

The thiol, the His-His motif, and the battle over Cu(II) in the relationship of CopM metallophore and OprC outer membrane protein

Aleksandra Hecel[1], Arian Kola[2], Daniela Valensin[2], Danuta Witkowska[3]*

[1] Faculty of Chemistry, University of Wroclaw, 50-383 Wroclaw, Poland

[2] Department of Biotechnology, Chemistry and Pharmacy, University of Siena, 53100 Siena, Italy

[3] Institute of Health Sciences, University of Opole, 45-060 Opole, Poland

*aleksandra.hecel2@uwr.edu.pl

Table S1 Stoichiometry, molecular formula and average m/z value for the species present in ESI-MS spectra of Cu(II) complexes with the studied ligands, M:L molar ratio = 1:1 in water:methanol 50:50 solution.

Molecular formula	*Ion signal*	*m/z*
**Ac-GACPNRMDAAAAAAAADHIMD-NH$_2$**		
$C_{83}H_{134}N_{28}O_{29}S_3$	$[L]^{2+}$	1042.95
$C_{83}H_{130}N_{28}O_{29}S_3Cu$	$[CuL]^{2+}$	1072.91
**Ac-EMMTPHHQDAIDMAEMALQKAEHPE-NH$_2$**		
$C_{122}H_{191}N_{35}O_{41}S_4$	$[L]^{3+}$	978.12
$C_{122}H_{191}N_{35}O_{41}S_4$	$[L]^{4+}$	733.84
$C_{122}H_{189}N_{35}O_{41}S_4Cu$	$[CuL]^{3+}$	998.76
$C_{122}H_{189}N_{35}O_{41}S_4Cu$	$[CuL]^{4+}$	749.32
**Ac-GMMGMHQGHGMMAMD-NH$_2$**		
$C_{64}H_{103}N_{21}O_{19}S_6$	$[L]^{2+}$	831.81
$C_{64}H_{102}N_{21}O_{19}S_6Na$	$[L+Na]^{3+}$	562.20
$C_{64}H_{97}N_{21}O_{19}S_6Cu$	$[CuL]^{2+}$	860.82
$C_{64}H_{96}N_{21}O_{19}S_6NaCu$	$[CuL+Na]^{3+}$	581.21

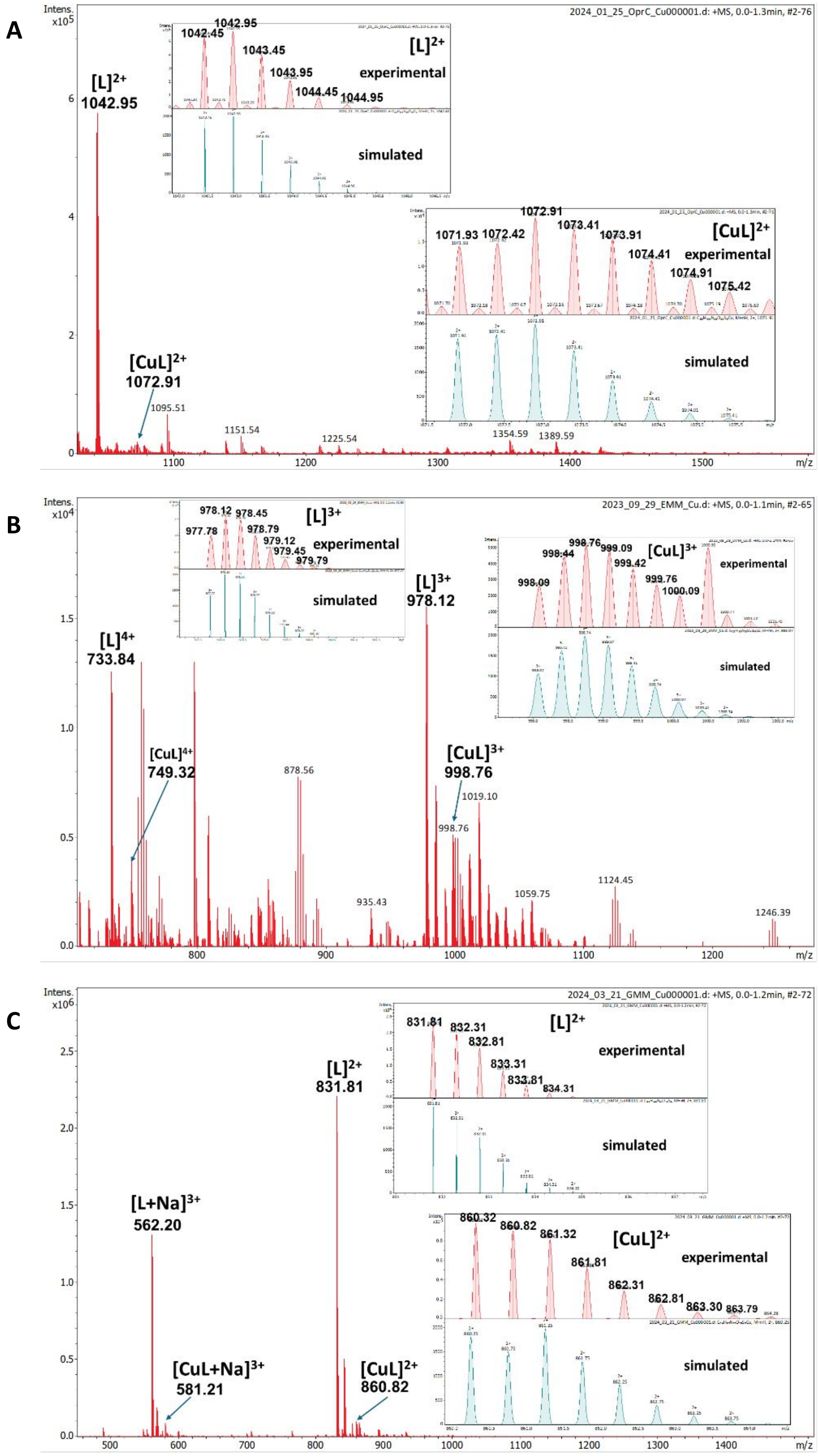

Figure S1 ESI-MS spectrum of A) Cu(II)-Ac-GACPNRMDAAAAAAAADHIMD-NH_2; B) Cu(II)-Ac-EMMTPHHQDAIDMAEMALQKAEHPE-NH_2; C) Cu(II)-Ac-GMMGMHQGHGMMAMD-NH_2; M:L molar ratio = 1:1 in water:methanol 50:50 solution. For chosen ligands and complexes a comparison of experimental and simulated signals were performed on ESI-MS spectra.

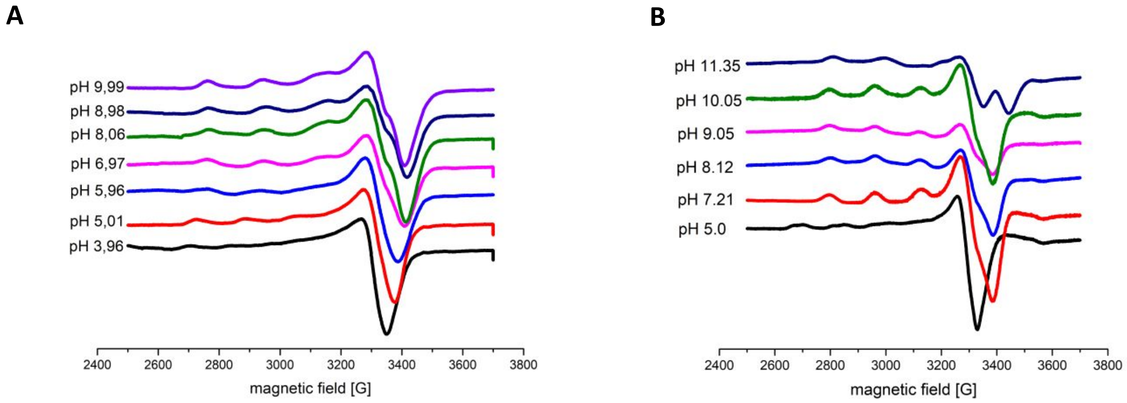

Figure S2 X-band EPR spectra of frozen solution (77 K) of A) Cu(II)-Ac-GACPNRMDAAAAAAAADHIMD-NH$_2$; B) Cu(II)-Ac-EMMTPHHQDAIDMAEMALQKAEHPE-NH$_2$ at different pH values; I=0.1 M (NaClO$_4$), metal:ligand molar ratio 1:1, [Cu(II)] = 1 mM. EPR parameters g_{II} and A_{II} for different pH values corresponding to the maximum concentrations of given complex species are collected at Table 2 in the main text. Due to the low solubility of the Cu(II)-Ac- GMMGMHQGHGMMAMD-NH$_2$ complex at concentration above 0.5 mM, the EPR technique was not used to complement UV-Vis and CD results. To observe clear hyperfine splitting in EPR spectra, the concentration of Cu(II) should be above 1mM. In this case, the Cu(II) binding modes at different pH were determined based on sufficient and converging UV-Vis and CD data.

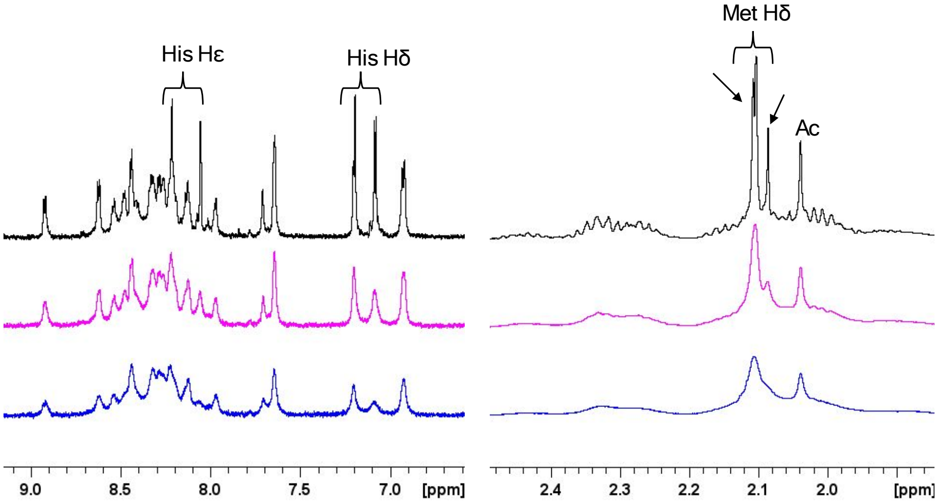

Figure S3 Superimposition of aromatic and aliphatic regions of 1D ^{1}H NMR spectra of Ac-$_{51}$EMMTPHHQDAIDMAEMALQKAEHPE$_{75}$-NH$_2$ (0.5 mM) in the absence (black) and presence of 0.1 (magenta) and 0.2 (blue) Cu(II) equivalents. Spectra were recorded at 288 K in 20 mM phosphate buffer, pH 7.4.

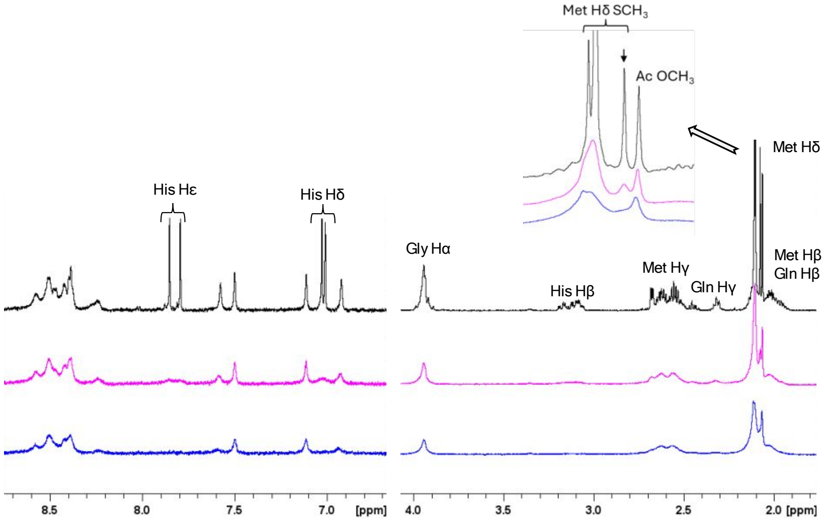

Figure S4 Superimposition of aromatic and aliphatic regions of 1D ^{1}H NMR spectra of Ac-$_{113}$GMMGMHQGHGMMAMD$_{127}$-NH$_2$ (0.5 mM) in the absence (black) and presence of 0.1 (magenta) and 0.2 (blue) Cu(II) equivalents. Spectra were recorded at 288 K in 20 mM phosphate buffer, pH 7.4.

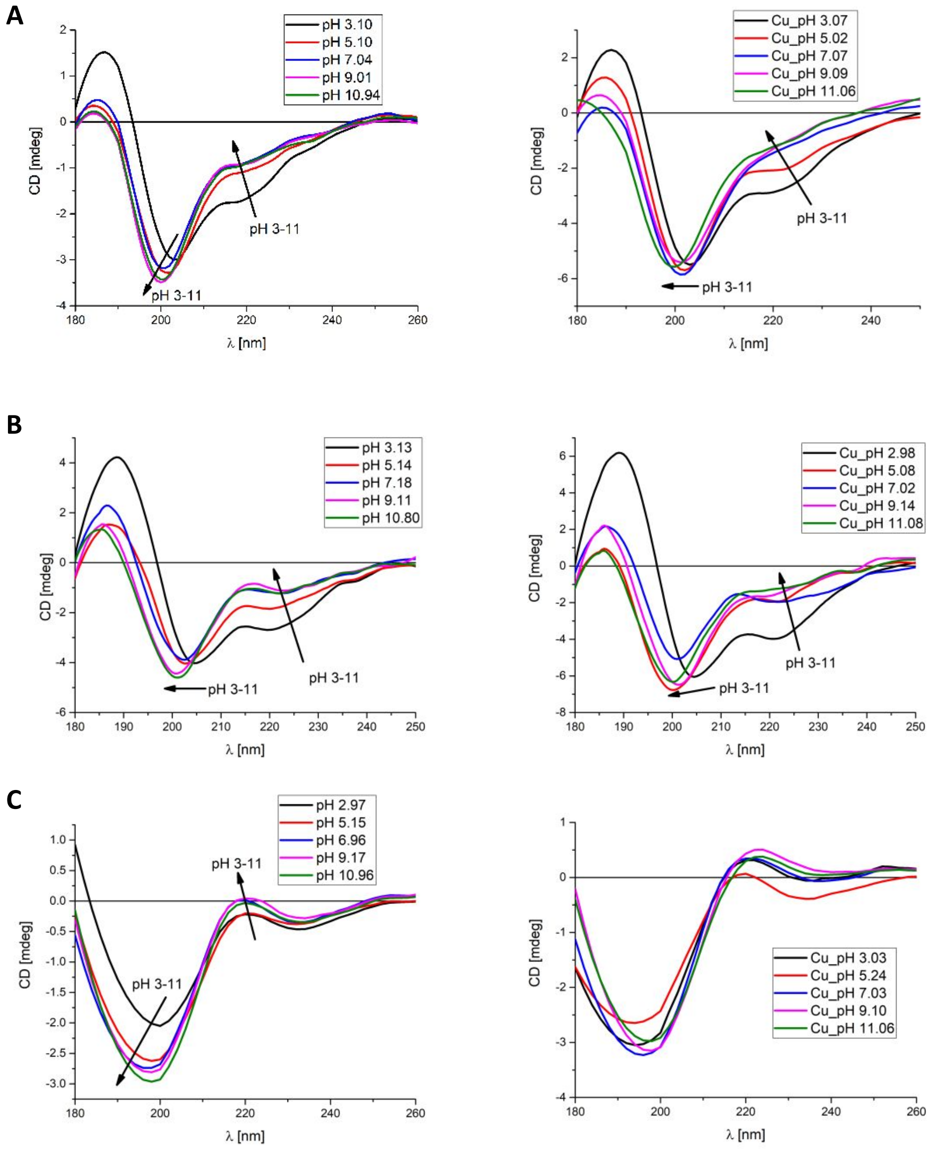

Figure S5 Far-UV CD spectra for apo ligand and Cu(II) complexes for A) Ac-GACPNRMDAAAAAAAADHIMD-NH_2, B) Ac-EMMTPHHQDAIDMAEMALQKAEHPE-NH_2 and C) Ac-GMMGMHQGHGMMAMD-NH_2 systems in aqueous solution of 4 mM $HClO_4$ with I = 0.1 M $NaClO_4$ in different pH values; optical path length = 0.1 mm. C_L = 0.1 mM.

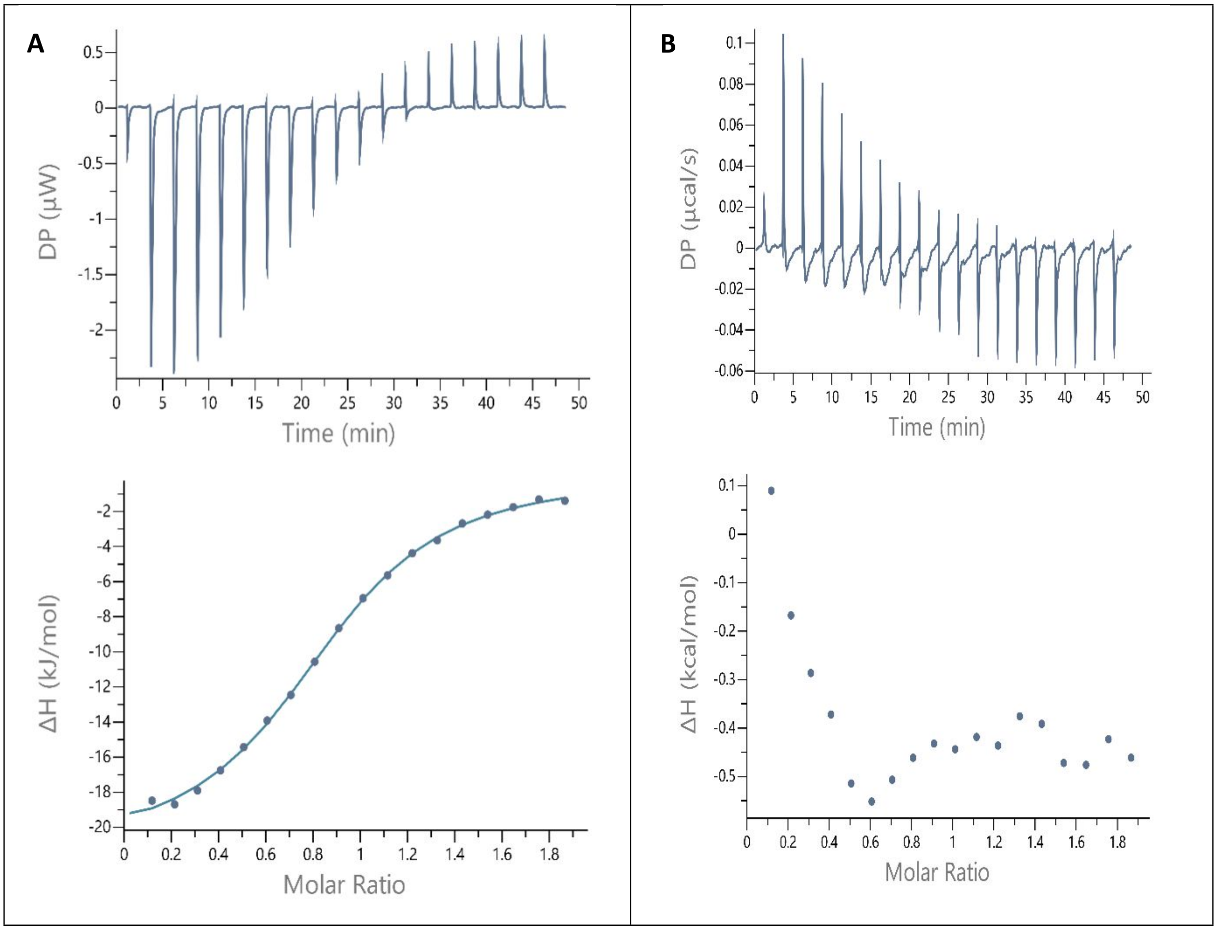
A
DP (μW)
Time (min)
ΔH (kJ/mol)
Molar Ratio
B
DP (μcal/s)
Time (min)
ΔH (kcal/mol)
Molar Ratio

C

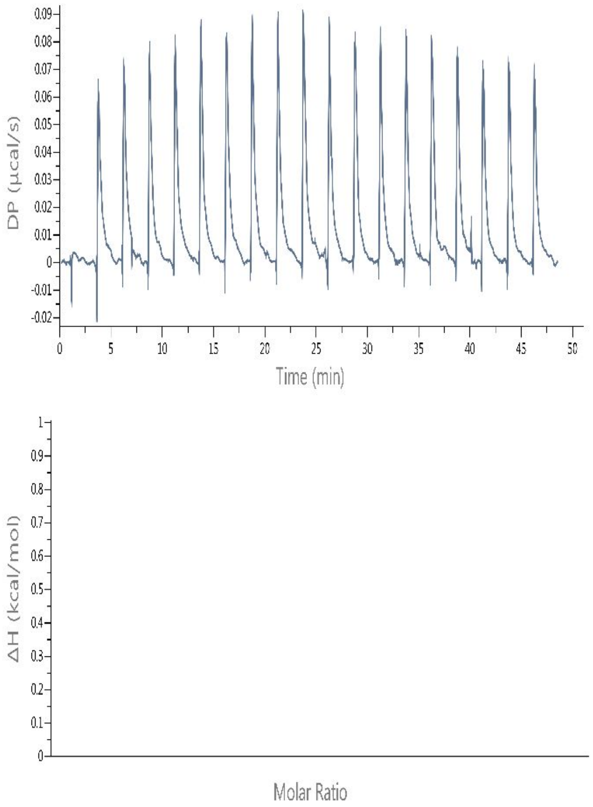

Figure S6 Representative ITC data (top) and corresponding thermodynamic signatures (bottom) shown for (A) the Ac-EMMTPHHQDAIDMAEMALQKAEHPE-NH_2 peptide (1.0 mM) titrated into Cu(II) in the presence of Ac-GACPNRMDAAAAAAAADHIMD-NH_2 at a 1:1 ratio, (B) Ac-GACPNRMDAAAAAAAADHIMD-NH_2 titrated into Cu(II) in the presence of the Ac-EMMTPHHQDAIDMAEMALQKAEHPE-NH_2 peptide at a 1:1 ratio and (C) Cu(II) titrated into a Caco buffer.
